# Stigmasterol Restores the Balance of Treg/Th17 Cells by Activating the Butyrate-PPARγ Axis in Colitis

**DOI:** 10.3389/fimmu.2021.741934

**Published:** 2021-10-06

**Authors:** Shuting Wen, Long He, Zhuotai Zhong, Runyuan Zhao, Senhui Weng, Hong Mi, Fengbin Liu

**Affiliations:** ^1^ The First Clinical College, Guangzhou University of Chinese Medicine, Guangzhou, China; ^2^ Department of Gastroenterology, The First Affiliated Hospital of Guangzhou University of Chinese Medicine, Guangzhou, China; ^3^ Baiyun Hospital of The First Affiliated Hospital of Guangzhou University of Chinese Medicine, Guangzhou, China; ^4^ Lingnan Medical Research Centre, Guangzhou University of Chinese Medicine, Guangzhou, China

**Keywords:** inflammatory bowel disease, stigmasterol, microbiota, butyrate, PPARγ, Treg/Th17 balance

## Abstract

Inflammatory bowel disease (IBD) is a chronic inflammatory disorder with gut microbiota disequilibrium and regulatory T (Treg)/T helper 17 (Th17) immune imbalance. Stigmasterol, a plant-derived sterol, has shown anti-inflammatory effects. Our study aimed to identify the effects of stigmasterol on experimental colitis and the related mechanisms. Stigmasterol treatment restored the Treg/Th17 balance and altered the gut microbiota in a dextran sodium sulfate (DSS)-induced colitis model. Transplantation of the faecal microbiota of stigmasterol-treated mice significantly alleviated inflammation. Additionally, stigmasterol treatment enhanced the production of gut microbiota-derived short-chain fatty acids (SCFAs), particularly butyrate. Next, human naïve CD4+ T cells sorted from IBD patients were cultured under Treg- or Th17-polarizing conditions; butyrate supplementation increased the differentiation of Tregs and decreased Th17 cell differentiation. Mechanistically, butyrate activated peroxisome proliferator-activated receptor gamma (PPARγ) and reprogrammed energy metabolism, thereby promoting Treg differentiation and inhibiting Th17 differentiation. Our results demonstrate that butyrate-mediated PPARγ activation restores the balance of Treg/Th17 cells, and this may be a possible mechanism, by which stigmasterol attenuates IBD.

## Introduction

Inflammatory bowel disease (IBD) is a chronic and relapsing form of intestinal inflammation that includes Crohn’s disease (CD) and ulcerative colitis (UC) (1). The incidence and prevalence of IBD continues to increase (2). IBD is a condition that may increase the risk of premalignant and malignant lesions with recurrent episodes of diarrhoea, abdominal pain, rectal bleeding, and fatigue, which severely impact patients’ quality of life (3). The immune response of the intestinal mucosa is associated with IBD, and an imbalance of regulatory T (Treg) cells and T helper 17 (Th17) cells can contribute to IBD pathogenesis.

Treg cells and Th17 cells perform specialized functions through distinct transduction pathways and restrict each other in terms of differentiation and function (4). Tregs have immunosuppressive properties that can also be present in the intestinal mucosa. Tregs are crucial to maintain immune homeostasis and can protect the host from an overactive immune response and tissue damage (5). In addition to inhibiting inflammation, Tregs can also regulate processes such as metabolic homeostasis and tissue repair (6). Th17 cells regulate host defence and produce inflammatory factors such as interleukin 17A (IL-17A), interleukin 17F (IL-17F), interleukin 21 (IL-21), and interleukin 22 (IL-22) (7). Similarly, regulating the Treg/Th17 balance can improve the intestinal inflammatory microenvironment and intestinal immune balance reconstitution (8).

Intestinal mucosal immunity and the gut microbiota are inextricably linked. Increasing evidence has shown that gut microbiota dysbiosis is related to the initiation of IBD (9), providing novel insight into IBD therapy. Intestinal mucosal immunity contributes to the balance of gut microbiota composition, while the gut microbiome and its metabolite short-chain fatty acids (SCFAs) serve as crucial factors of the mucosal immune response (10, 11). Similarly, specific intestinal flora can influence Treg and Th17 cell differentiation (12, 13). Imbalance of the gut microbiota and SCFAs can provoke a Treg/Th17 imbalance, leading to the onset and progression of IBD (14, 15).

Currently, IBD is incurable and the treatments include 5-aminosalicylic acid (5- ASA), steroids, antimicrobials, immunosuppressive agents and TNF blockers. However, the high reoccurrence rates and adverse side effects of these treatment have necessitated the development of novel therapeutic approaches. Stigmasterol is a phytosterol that exerts anti‐inflammatory, antioxidative, and antitumour properties and improves metabolism (16, 17). Stigmasterol is also reported to be the active ingredient in common Chinese herbal compound for the treatment of IBD (18, 19). Previous studies have demonstrated that stigmasterol can significantly lower the expression of cyclooxygenase-2 (COX-2) and improve the colonic inflammation score, thereby ameliorating colitis (20). Nevertheless, the underlying mechanism has not yet been completely clarified.

This study aimed to explore the effects of stigmasterol against IBD and obtain further insight into their mechanisms of action. First, we used a dextran sodium sulfate (DSS)-induced colitis mouse model to validate the efficacy of stigmasterol against IBD. Next, we conducted faecal microbiota transplantation (FMT) and induced intestinal flora disorders to identify the critical role of the intestinal flora and its metabolite SCFAs in alleviating colitis by stigmasterol. *In vivo* experiments showed that stigmasterol decreased DSS-induced inflammatory responses and regulated the Treg/Th17 balance. The efficacy of stigmasterol depended on the gut microbiome and its metabolites. Finally, we explored the underlying mechanism by which metabolites affect the differentiation of Treg and Th17 cells using *in vitro* experiments, which provided innovative insight into treating IBD.

## Methods

### Mice

Wild-type (WT) male C57BL/6 mice were purchased from Guangdong Medical Laboratory Animal Center (Guangdong, China). They were bred in a specific pathogen-free (SPF) environment at 22 ± 2°C and a humidity of 55 ± 10% on a 12-hour light/dark cycle. The mice were aged 8–10 weeks and weighed 25–30 grams at the beginning of the experiments. Animal care and management protocols were performed according to the Institutional Animal Ethics Committee of the First Affiliated Hospital of Guangzhou University of Chinese Medicine (ethics no. TCMF1-2019063).

### DSS-Induced Colitis and Treatments

Mice were administered 2.5% DSS (w/v; 36,000–50,000 M.W.; MP Biomedicals, Santa Ana, California, USA) in water for 7 days to induce experimental colitis and then administered regular water for 3 days. Stigmasterol (400 mg/kg/d) was provided to stigma + DSS group mice orally by gavage for 10 days. The stool consistency, faecal blood, and weight loss of each mouse were assessed daily. The DAI score is the mean of the three parameters (**Supplementary Table S1**) (21). On day 10, fresh faeces were collected from the mice for FMT experiments and 16S rRNA sequencing.

### Gut Microbiota Depletion and FMT

The mice were administered an ABX cocktail orally by gavage for 5 days to deplete the gut microbiota. The cocktail comprised amphotericin (1 mg/mL), neomycin (10 mg/mL), vancomycin (50 mg/mL) and metronidazole (100 mg/mL) (22). Fresh faeces were collected from donor mice (from the DSS and stigma + DSS groups) and resuspended in PBS (100 mg/mL). The suspension was vortexed thoroughly for 30 s before centrifugation. Next, the supernatant was intragastrically administered to antibiotic-treated mice once a day for 5 days.

### Histopathology and Immunofluorescence

Colon tissues were cleaned with PBS and fixed in 4% paraformaldehyde. Next, they were embedded in paraffin, sectioned and stained with haematoxylin and eosin (H&E, magnification: 40× and 200×). Histological sections were blindly observed by an experienced pathologist. Histological scores, including the crypt structure, inflammation, mucosal thickening, crypt abscesses and goblet cell exhaustion, were evaluated (**Supplementary Table S2**). Immunofluorescence staining for PPARγ (red) and DAPI (blue) in colon tissue sections was also performed according to the manufacturer’s instructions.

### Enzyme-Linked Immunosorbent Assay (ELISA)

Distal colon tissues were homogenized, and proteins were extracted. A BCA kit (Beyotime, Beijing, China) was used to determine the protein concentrations. According to the manufacturer’s instructions (Dogesce, Beijing, China; Andygene Biotechnology Co., Ltd, Beijing, China), the concentrations of the inflammatory cytokines IL-6, IL-1β, TNF-α and serum IL-10, TGF-β and IL-17A levels were then measured using ELISA kits. The absorbance at 450 nm was measured using a microplate reader (Thermo Scientific, Waltham, MA, USA).

### 16S rRNA Sequencing and Bioinformatic Analysis

Genomic DNA was extracted from faecal samples and quantified. Quality genomic DNA samples were then used for PCR amplification. The V4 region of the 16S rRNA gene was amplified using the 515F-806R primer. PCR was performed in a PCR thermocycler (GeneAmp 9700; Applied Biosystems, France). A HiSeq 2500 system (Illumina, San Diego, CA, USA) was used for sequencing. For 16S analysis, *QIIME (version 1.9.1)* was used to demultiplex and quality-filter the raw FASTQ files. Next, operational taxonomic units (OTUs) were generated and clustered using a 97% threshold and *Usearch (version 7.1)*. After aligning with the *gold database (v20110519)*, *UCHIME (4.2.40)* was used to filter out chimaeric sequences, and usearch_global was used to quantify the OTU abundances for each sample. For taxonomic analysis of each representative OTU, the *Greengenes database (v201305)* was used based on the *RDP classifier (Version 2.2)* with a 0.8 confidence value. OTUs were then assigned to different hierarchical levels and taxonomic relative abundance profiles were summarized. Alpha diversity was studied using diversity (Shannon) and richness (Chao, ace, sobs) indexes.

### Targeted Quantitative Analysis of Faecal SCFA Contents

The contents of faecal SCFAs were assessed using gas chromatography coupled with mass spectrometry (Agilent 7890B/MSD 5977A; Wilmington, DE, USA). Mixtures of acetate, propionate, butyrate, isobutyrate, valerate, and isovalerate were used as standards, which were acquired from Sigma-Aldrich (St. Louis, MO, USA).

### Molecular Docking

The crystal structure of the protein was obtained from the Protein Data Bank (PDB) database (https://www.rcsb.org/). For the target preparation, the original ligands and water molecules of the protein were deleted, and hydrogen was added to polar groups *via PyMOL 2.3.2*. For ligand preparation, the chemical structures were retrieved from the DrugBank database (https://www.drugbank.ca). Using *AudoDock MGL Tools 1.5.6*, atomic partial charges were added, and the torsional root of the ligands was chosen. The molecular docking calculations were performed using *Autodock Vina 1.1.2*. The docking results were analysed and visualized using *PyMOL 2.3.2*.

### Isolation of Mononuclear Cells From the Spleen, Mesenteric Lymph Nodes (MLNs) and Colon

Single-cell suspensions from mouse spleens and MLNs were prepared using a 100-μm cell strainer. After centrifugation, red blood cells (RBCs) were lysed with ACK buffer. Colonic lamina propria mononuclear cells (LPMCs) were extracted as previously described (23). Next, the cells were washed with staining buffer and used for flow cytometry analysis.

### Flow Cytometry

Treg and Th17 cells were identified using flow cytometry. Lymphocytes were stained with anti-CD4 FITC and anti-Foxp3 PE antibodies to identify Treg cells. To identify Th17 cells, lymphocytes were first stimulated in 5% carbon dioxide at 37°C for 6–8 hours using a Cell Stimulation Cocktail (plus protein transport inhibitors), which contains phorbol ester, phorbol 12-myristate 13-acetate (PMA), ionomycin, brefeldin A and monensin. The lymphocytes were then stained with anti-CD4 FITC and anti-IL-17 PE antibodies. The percentages of CD4+Foxp3+ (Treg) cells and CD4+IL-17+ (Th17) cells were then detected by flow cytometry. The data were analysed using FlowJo V.10 software. All the reagents were purchased from Tonbo Biosciences (San Diego, CA, USA).

### Human Naïve CD4+ T Cell Isolation

Blood from IBD patients was collected after informed written consent was obtained. Peripheral blood mononuclear cells (PBMCs) were isolated from diluted blood by density-gradient centrifugation using lymphocyte separation solution (TBD, Tianjin, China). Naïve CD4^+^ T cells were then separated using the EasySep™ Human Naïve CD4^+^ T Cell Isolation Kit (Stem Cell Technologies, London, UK). All human blood samples were obtained from the First Affiliated Hospital of Guangzhou University of Chinese Medicine, China. This study was approved by the Medical Ethics Committee of the Academic Medical Center of Guangzhou University of Chinese Medicine (ethics no. JY[2021]025) and was registered in the Chinese Clinical Trial Registry (ChiCTR) (registration no. ChiCTR2100043238; registration date: 2021-02-09).

### *In Vitro* Treg and Th17 Cell Differentiation

Naïve CD4+ T cells were cultivated in a 96-well plate (1 × 10^5^ cells/well) containing plate-bound anti-CD3 antibody (2.5 µg/ml) and anti-CD28 antibody (5 µg/ml) (Tonbo Biosciences). To induce Treg cell differentiation, TGF-β1 (3 ng/ml) and IL-2 (2 ng/ml) were added. To induce Th17 cell differentiation, recombinant human TGF-β1 (3 ng/ml), IL-23 (25 ng/ml), anti-IL-4 antibody (5 µg/ml), and IFN-γ antibody (5 µg/ml) (PeproTech) were added. Naïve CD4+ T cells were treated with either butyrate alone (0.2 mM) or both butyrate (0.2 mM) and GW9662 (10 µM) for 5 days. Next, the cells were used for flow cytometry analysis, western blotting, glycolytic rate analysis and a mitochondrial stress assay.

### Western Blotting

RIPA buffer (Beyotime) was used to prepare cell lysates. After quantification of the protein concentration using a BCA kit, the proteins were dissolved *via* SDS-PAGE and transferred to polyvinylidene fluoride (PVDF) membranes. After blocking and incubation with the primary antibody, the membrane was then probed with secondary antibodies. The protein bands were detected and visualized using a ChemiDoc Imaging System (Bio-Rad). Antibodies against PPAR gamma (Abcam, ab178860), HIF-1α (Abcam, ab216842), and β-Actin (Abcam, ab8226) were used.

### Measurement of the OCR and GlycoPER

OCR and glycoPER were measured using a Seahorse XF24 Extracellular Flux Analyser (Seahorse Bioscience, Agilent Technologies, Santa Clara, CA). A Seahorse XF Cell Mito Stress Test Kit (103015-100; Seahorse Bioscience) and Seahorse XF Glycolytic Rate Assay Kit (103344-100; Seahorse Bioscience) were also used. T cells (3×10^5^/well) were seeded in 24-well Cell-Tak-coated (22.4 μg/ml, Corning) Seahorse culture plates for analysis. For OCR measurement, 1.5 μM oligomycin, 0.5 μM FCCP and 0.5 μM antimycin-A/rotenone were added sequentially. To measure the glycoPER and mitoOCR/glycoPER ratio, 0.5 μM Rot/AA and 50 mM 2-DG were added sequentially.

### Statistical Analysis

GraphPad Prism 8.0 (GraphPad Software, San Diego, USA) was used to analyse the data, which are presented as means ± SD. Unpaired two-tailed Student’s t-test or one-way analysis of variance (ANOVA) was performed for statistical analyses. Survival differences were assessed using the log-rank test. P values were defined as follows: * P < 0.05, ** P < 0.01, *** P < 0.001; n.s., not significant (P > 0.05).

## Results

### Stigmasterol Delays the Onset of Colitis

**Figure 1:**
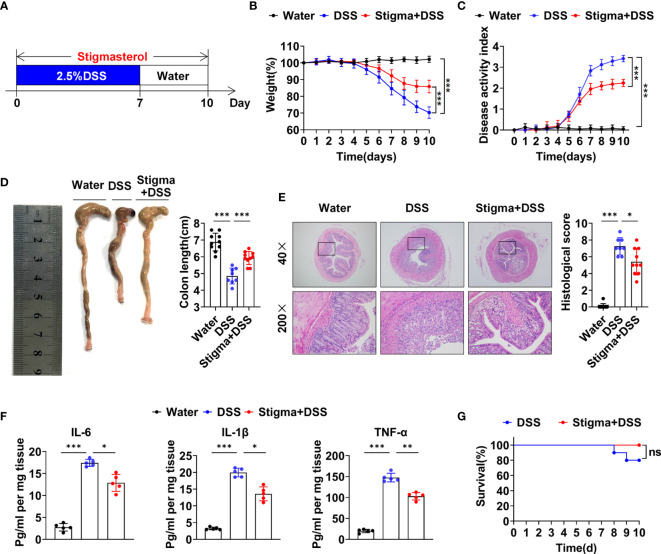
Stigmasterol treatment attenuates DSS-induced colitis. **(A)** Schematic of the pharmacodynamic study of stigmasterol. C57BL/6 mice were fed drinking water supplemented with 2.5% DSS to induce acute colitis. The stigma + DSS group was orally gavaged once daily with stigmasterol for 10 days. **(B, C)** Body weights **(B)** and DAI scores **(C)** of the water, DSS and stigma + DSS groups (n = 10 per group). **(D)** Macroscopic appearance and length of the colon (n = 10 per group). **(E)** Representative microscopy images (40× and 200× magnification) of H&E-stained colon tissues and corresponding histological scores (n = 10 per group). **(F)** Analysis of inflammatory cytokine expression in distal colon tissues. The IL-6, IL-1β and TNF-α cytokine levels in colon tissue homogenates from the water, DSS and stigma + DSS groups were measured by ELISA (n = 5 per group). **(G)** Survival curves of the DSS and stigma + DSS groups (n = 10 per group). **(B–G)** The data are representative of three independent experiments. *P < 0.05, **P < 0.01, ***P < 0.001; n.s., not significant (P > 0.05).

First, we explored the protect effect of stigmasterol on colitis. The workflow used to study the pharmacodynamics of stigmasterol is illustrated in **Figure 1A**. Compared with the DSS group, the mouse body weight was increased (**Figure 1B**), the DAI score was decreased (**Figure 1C**), and shortening of the colon was alleviated (**Figure 1D**) in the stigma + DSS group. Furthermore, stigmasterol appreciably reduced the colon pathohistological score (**Figure 1E**) and downregulated the expression of inflammatory factors, including IL-6, IL-1β and TNF-α, in DSS-treated mice (**Figure 1F**). However, no noticeable difference in mortality was observed (**Figure 1G**). The above results suggested that stigmasterol prevents DSS colitis and may protective against IBD.

### Stigmasterol Regulates the Treg/Th17 Balance

**Figure 2:**
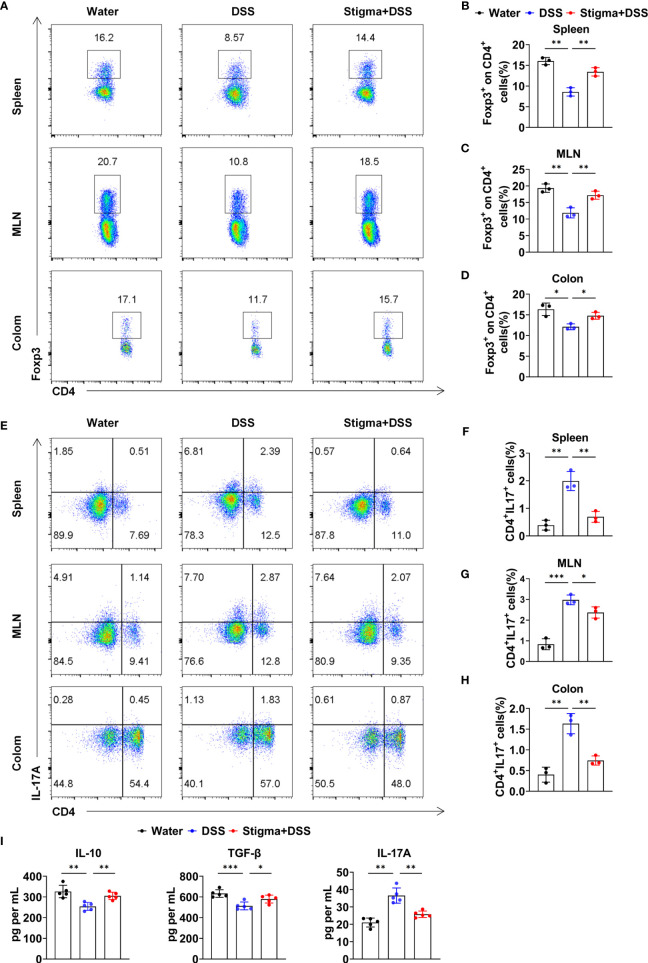
Stigmasterol treatment plays a role in regulating the Treg/Th17 balance in the spleen, MLNs and colon. **(A–D)** Flow cytometry analysis **(A)** and the average number of CD4^+^Foxp3^+^ (Treg)/CD4^+^ T cells in the spleen **(B)**, MLNs **(C)** and colon **(D)**. **(E–H)** Flow cytometry analysis **(E)** and the average number of CD4^+^IL17A^+^(Th17) cells in the spleen **(F)**, MLNs **(F)** and colon **(H)**. **(A–H)** n = 3 mice per group. **(I)** Serum IL-10, TGF-β and IL-17A were measured by ELISA. n = 5 mice per group. **(A–I)** The data are representative of three independent experiments. *P < 0.05, **P < 0.01, ***P < 0.001.

We next explored whether stigmasterol protects against colitis by regulating the Treg/Th17 balance. Flow cytometric analyses demonstrated that the proportion of Tregs among gated CD4^+^ T cells in the spleen, MLNs and colon was reduced in DSS colitis. However, stigmasterol increased the Treg proportion (**Figures 2A–D**). Similarly, DSS-induced colitis mice exhibited a higher percentage of Th17 cells than controls, and stigmasterol reduced the percentage of Th17 cells (**Figures 2E–H**). Additionally, stigmasterol increased the expression of IL-10 and TGF-β but downregulated the expression of IL-17A (**Figure 2I**). These findings strongly suggested that stigmasterol promotes Treg cell development and inhibits the Th17 cell response in DSS-induced colitis mice.

### Stigmasterol Alters the Gut Microbiota, and Therapeutic FMT Alleviates Colitis

**Figure 3:**
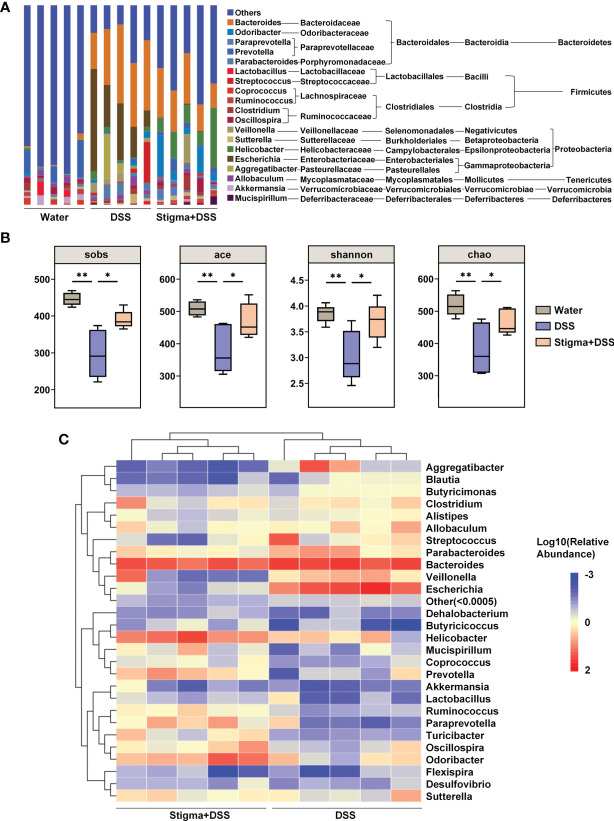
Stigmasterol treatment significantly alters the gut microbiota diversity and composition. **(A)** Analysis of the faecal microbiota composition of mice in the water, DSS and stigma + DSS groups at the genus level (relative abundance). **(B)** Alpha diversity boxplot (Sobs, ACE, Shannon and Chao indexes). *P < 0.05, **P < 0.01. **(C)** Heatmap of most differentially abundant features at the genus level. A change from blue to red represents low to high abundance. **(A–C)** n = 5 samples per group. Each symbol represents an individual mouse.

Since the gut microbiota plays important roles in IBD pathogenesis, we next determined whether stigmasterol influences the gut microbiota. At the genus level, stigmasterol increased the composition of *Ruminococcus*, *Prevotella*, *Paraprevotella*, *Helicobacter*, *Odoribacter*, *Clostridium_IV*, and *Clostridium_XlVa*. Additionally, stigmasterol decreased the abundances of *Streptococcus*, *Escherichia*, *Enterococcus* and *Allobaculum* (**Figure 3A** and **Supplementary Table S3**). Different diversity indexes (Sobs, Shannon, ACE, and Chao) showed similar trends: stigmasterol improved the diversity of intestinal microflora (**Figure 3B**). Clustering analysis of OTUs revealed the differences in the microbial community among the three groups (**Supplementary Figure S1**). The dominant bacteria in the stigma+DSS group included *Helicobacter*, *Odoribacter*, *Prevotella*, *Oscillospira*, *Paraprevotella*, *Turicibacter*, *Ruminococcus*, *Butyricicoccus*, *Ruminococcaceae*, and *Paraprevotellaceae* (**Supplementary Figure S2**). The differences in the faecal microbiota composition are presented in a heat map (**Figure 3C**). Additionally, stigmasterol FMT treatment alleviated colitis (**Supplementary Figure S3**) and restored the Treg/Th17 balance (**Supplementary Figure S4**). Because the trend of Treg/Th17 cells in the colon was consistent with that in the spleen and MLNs, we detected the Treg/Th17 cells in only the spleen and MLNs in the FMT experiment.

### Stigmasterol Promotes the Production of SCFAs in Faeces

**Figure 4:**
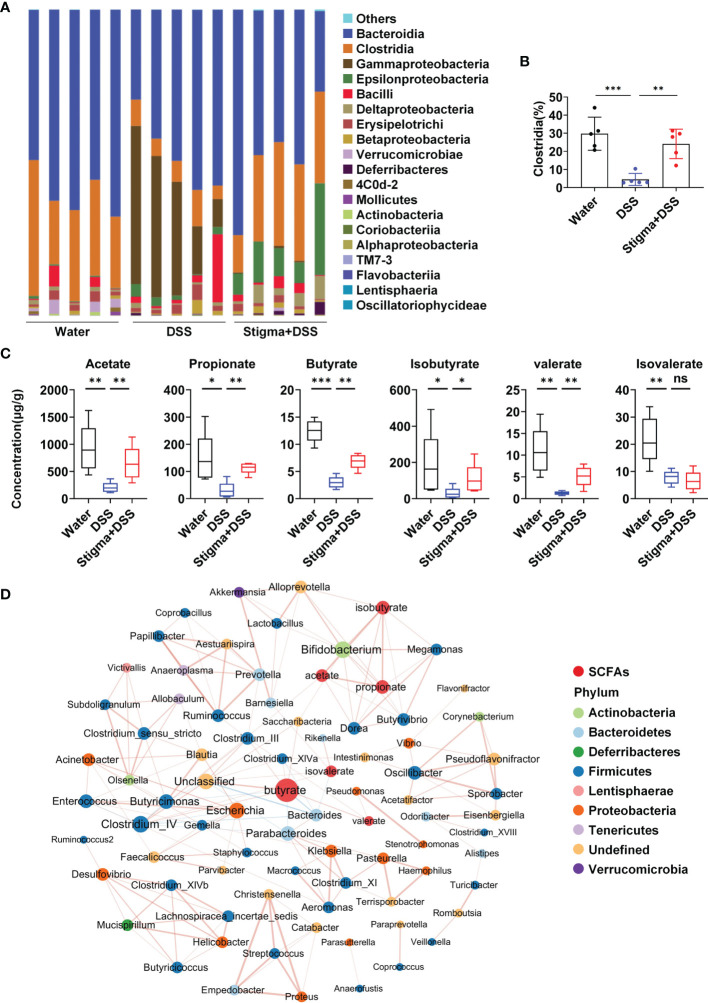
Stigmasterol treatment increases the production of microbial metabolite SCFAs. **(A)** Analysis of the faecal microbiota composition of mice in the water, DSS and stigma + DSS groups at the class level (relative abundance). **(B)** Stigmasterol treatment increased the relative abundance of Clostridia in the gut. **(C)** SCFA concentrations in the faeces of the stigma + DSS and DSS groups. **(A–C)** n = 5 mice per group. *P < 0.05, **P < 0.01, ***P < 0.001; n.s., not significant (P > 0.05). **(D)** Pearson correlation network showing relationships among the contents of SCFAs and abundance of bacterial genera in the water, DSS and stigma + DSS groups (|cor| > 0.7). The SCFAs included acetate, propionate, butyrate, isobutyrate, valerate and isovalerate. The colours of the nodes representing the bacterial genera indicate the phylum for that genus, the size of each node is proportional to the abundance of the bacterial community, and red indicates SCFAs. Each edge represents interactions among SCFAs and bacterial genera, red lines indicate positive correlations, and blue lines indicate negative correlations.

Then we analysed the faecal microbiota composition of each group at the class level (**Figure 4A** and **Supplementary Table S4**). We found that stigmasterol treatment increased the composition of Clostridia (**Figure 4B**), which is closely related to the production of SCFAs (24). Quantitative analysis of the contents of specific SCFAs demonstrated that the concentrations of SCFAs in faeces decreased in DSS-induced colitis mice. Additionally, stigmasterol treatment markedly increased the acetate, propionate, butyrate, isobutyrate and valerate levels in the faeces of DSS-induced colitis mice (**Figure 4C**). Pearson correlation-based network analysis revealed that the butyrate level was most strongly correlated with the abundance of certain species in the gut microbiota (**Figure 4D** and **Supplementary Table S5**). The abundance of *Clostridia* in the gut was positively correlated with the butyrate concentrations in faeces (**Supplementary Figure S5**). These results further suggested that stigmasterol supplementation results in a change in the intestinal microflora and affects the levels of SCFAs, particularly butyrate.

### Butyrate Restores the Treg/Th17 Balance in a PPARγ-Dependent Manner

**Figure 5:**
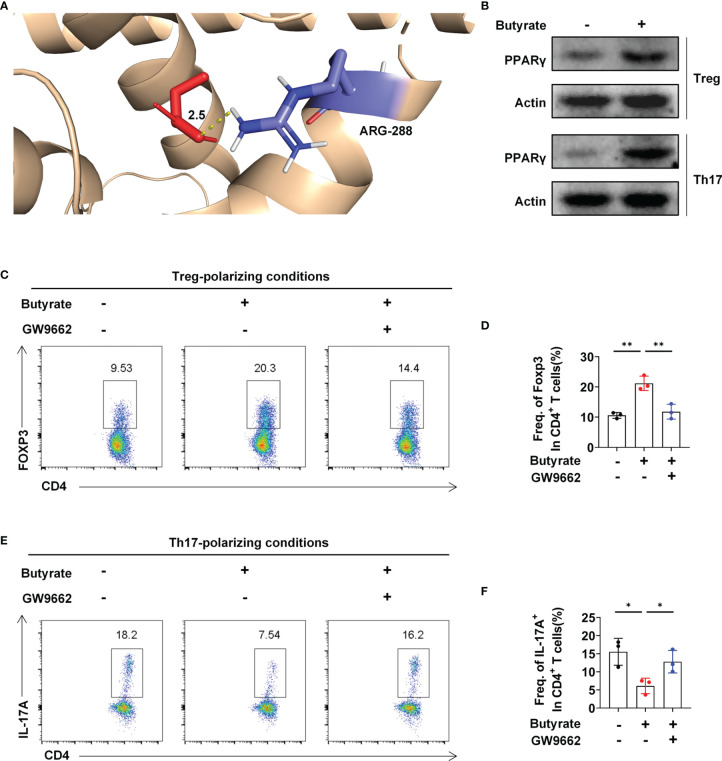
Butyrate regulates the Treg/Th17 balance by targeting PPARγ. **(A)** 3D diagrams of the molecular docking of butyric acid to PPARγ. **(B)** Western blot analysis of PPARγ levels in Treg and Th17 cells in the presence or absence of butyrate. **(C)** Flow cytometry analysis of FoxP3 expression in CD4^+^ T cells was performed. **(D)** The average number of Foxp3^+^ cells among CD4^+^ T cells. **(E)** Flow cytometry analysis of IL-17A^+^ expression in the CD4^+^ T-cell subset was performed. **(F)** The average number of IL-17A^+^ cells among CD4^+^ T cells. **(C–F)** n = 3 per group. The data are representative of three independent experiments. *P < 0.05, **P < 0.01.

Next, we explored the mechanism underlying butyrate-mediated Treg and Th17 cell differentiation *in vitro*. Molecular docking analysis suggested that butyrate directly interacted with PPARγ, a key target for IBD therapy which was upregulated by stigmasterol treatment (**Supplementary Figure S6**). Butyrate might bind to the active sites of PPARγ at Arg-288 (**Figure 5A**). Additionally, the expression of PPARγ in Treg and Th17 cells was upregulated after butyrate intervention (**Figure 5B**). To confirm that PPARγ activation is important for Treg and Th17 cell differentiation by butyrate, we used GW9662 to specifically inhibit PPARγ activity. Further analysis indicated that butyrate increased the expression of Treg cells; however, the promotion of Treg cell differentiation was inhibited after GW9662 treatment (**Figures 5C, D**). Consistently, butyrate reduced Th17 cells, and the inhibition of Th17 cell differentiation was attenuated after GW9662 treatment (**Figures 5E, F**).

### Butyrate Switches Metabolism From Glycolysis to Oxidative Phosphorylation (OXPHOS) in a PPARγ-Dependent Manner

**Figure 6:**
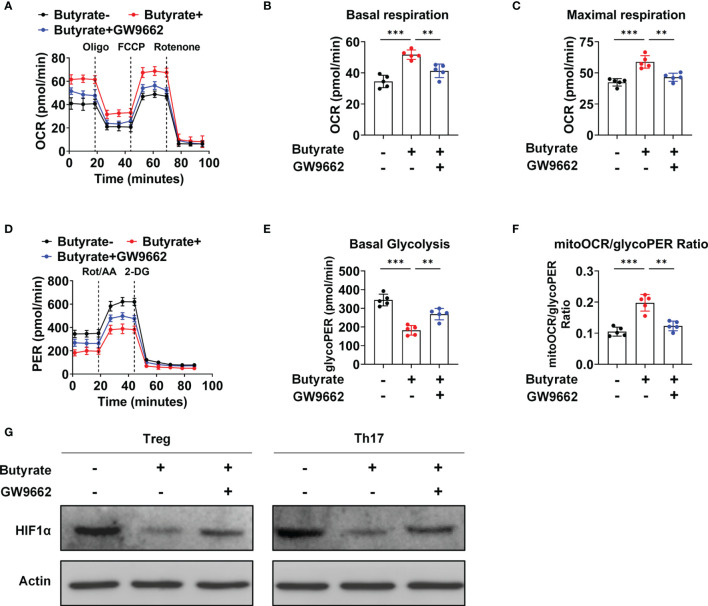
Butyrate mediates a metabolic shift from glycolysis to OXPHOS in IBD patients. **(A)** The OCR of cultured naïve CD4+ T cells (sorted from IBD patients) under Treg-polarizing conditions on day 5, as measured by Seahorse analysis. **(B, C)** The basal OCR **(B)** and maximal OCR **(C)** on day 5 of culture. **(D)** The extracellular acidification rate of naïve CD4+ T cells (sorted from IBD patients) under Th17-polarizing conditions, as assessed by Seahorse analysis. **(E, F)** Basal level of glycolysis **(E)** and the mitoOCR/glycoPER ratio **(F)** on day 5 of culture. **(A–F)** n = 5 per group. The data are representative of three independent experiments. **P < 0.01, ***P < 0.001. **(G)** Western blots showing the effect of different treatments on the HIF1-α levels.

Seahorse analyses demonstrated that butyrate supplementation increased the basal and maximal oxygen consumption rates (OCRs) in naïve CD4+ T cells under Treg-polarizing conditions; however, the OCR was decreased after GW9662 treatment (**Figures 6A–C**). This finding indicated that butyrate increased OXPHOS in a PPARγ-dependent manner, thereby promoting Treg cell differentiation. Simultaneously, under Th17 polarizing conditions, the level of glycolysis was decreased, and the mitochondrial OCR (mitoOCR)/proton efflux rate from the glycolysis (glycoPER) ratio was increased under butyrate treatment, whereas GW9662 treatment increased the basal level of glycolysis and decreased the mitoOCR/glycoPER ratio (**Figures 6D–F**). Hypoxia inducible factor-1α (HIF-1α) is critical for glycolytic metabolism (25). Butyrate supplementation reduced the expression of HIF-1α under Treg- and Th17-polarizing conditions, whereas GW9662 treatment increased the expression of HIF-1α (**Figure 6G**). The results suggested that butyrate promotes the metabolic transition of glycolysis to OXPHOS by activating PPARγ, thereby inducing T-cell differentiation into Treg cells but not Th17 cells.

### Stigmasterol-Induced Mitigation of Colitis Depends on the Intestinal Microflora

**Figure 7:**
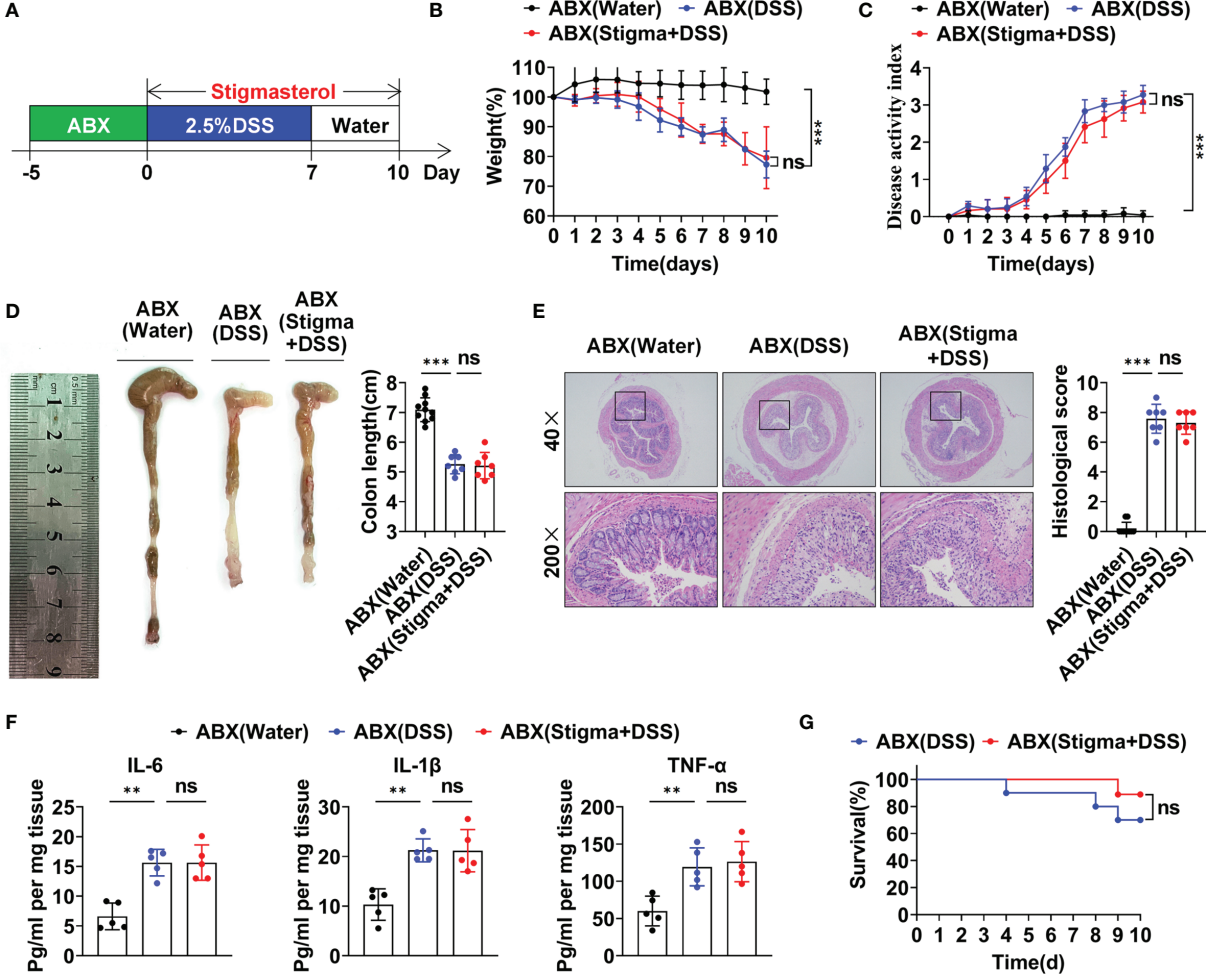
The gut microbiota plays a significant role in the protective effect of stigmasterol against DSS-induced colitis. **(A)** WT mice were pretreated with ABXs to deplete the gut microbiota. Next, the mice received 2.5% DSS. The ABX (stigma + DSS) group was orally gavaged once daily with stigmasterol from day 0 to day 10. **(B, C)** The body weights **(B)** and DAI scores **(C)** of the ABX (water) (black dotted line), ABX (DSS) (blue dotted line) and ABX (stigma + DSS) groups (red dotted line) (n = 10 per group). **(D)** Macroscopic appearance and length of the colon (n = 10 per group). **(E)** Representative microscopy images (40× and 200× magnification) of H&E-stained colon tissues and corresponding histological scores (n = 10 per group). **(F)** Analysis of inflammatory cytokine expression in distal colonic tissues. IL-6, IL-1β and TNF-α cytokine levels in colon tissue homogenates from the ABX (water), ABX (DSS) and ABX (stigma + DSS) groups were measured by ELISA (n = 5 per group). **(G)** Survival curves of the ABX (DSS) group and ABX (stigma + DSS) group (n = 10 per group). **(B–G)** Data are representative of three independent experiments. **P < 0.01, ***P < 0.001; n.s., not significant (P > 0.05).

**Figure 8:**
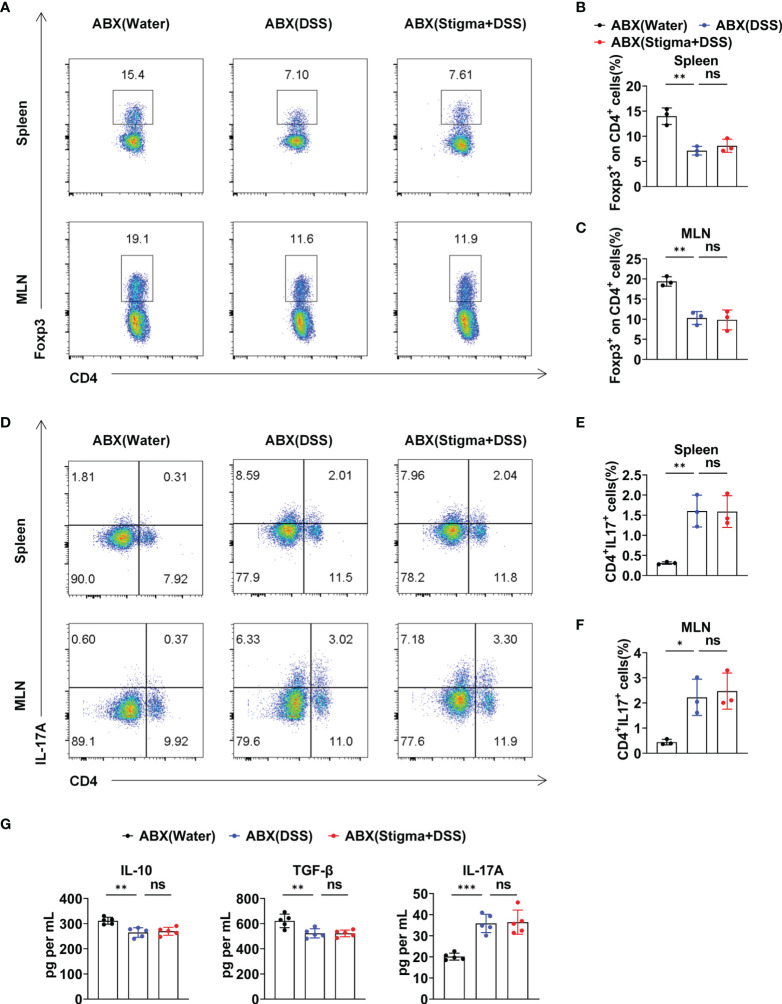
Stigmasterol regulates the Treg/Th17 balance in a gut microbiota-dependent manner. **(A)** The number of Treg cells in the spleen and MLNs in the ABX (water), ABX (DSS) and ABX (stigma + DSS) groups was analysed by flow cytometry. **(B, C)** Average number of CD4^+^Foxp3^+^ (Treg) cells among the CD4^+^ T-cell subsets in the spleen **(B)** and MLNs **(C)**. **(D)** The number of Th17 cells in the spleen and MLNs in the ABX (water), ABX (DSS) and ABX (stigma + DSS) groups was analysed by flow cytometry. **(E, F)** Bar charts showing the percentage of CD4^+^IL17A^+^(Th17) cells in the spleen **(E)** and MLNs **(F)**. **(A–F)** n = 3 mice per group. **(G)** Serum concentration of IL-10, TGF-β and IL-17A. n = 5 mice per group. **(A–G)** The data are representative of three independent experiments. *P < 0.05, **P < 0.01, ***P < 0.001; n.s., not significant (P > 0.05).

The aforementioned results revealed that stigmasterol restores the Treg/Th17 balance to ameliorate intestinal inflammation by modulating the intestinal microflora and its metabolite SCFAs. To further observe the role of the intestinal microflora, the mice were pretreated with an ABX cocktail to deplete the intestinal flora and then treated with stigmasterol (**Figure 7A**). Interestingly, when the gut microbiota was suppressed, the therapeutic effects of stigmasterol were abolished. Weight loss (**Figure 7B**), the DAI score (**Figure 7C**), the colon length (**Figure 7D**), the colonic histopathological score (**Figure 7E**), and the mortality rate (**Figure 7G**) were not substantially different between the ABX (DSS) and the ABX (stigma+DSS) groups. Similarly, no significant differences were found in the IL-6, IL-1β, or TNF-α (**Figure 7F**) levels or number of Treg cells (**Figures 8A–C**) or Th17 cells (**Figures 8D–F**). The serum IL-10, TGF-β, or IL-17A levels showed no apparent difference (**Figure 8G**). Overall, these results indicated that the gut flora is important in the therapeutic efficacy of stigmasterol.

## Discussion

**Figure 9:**
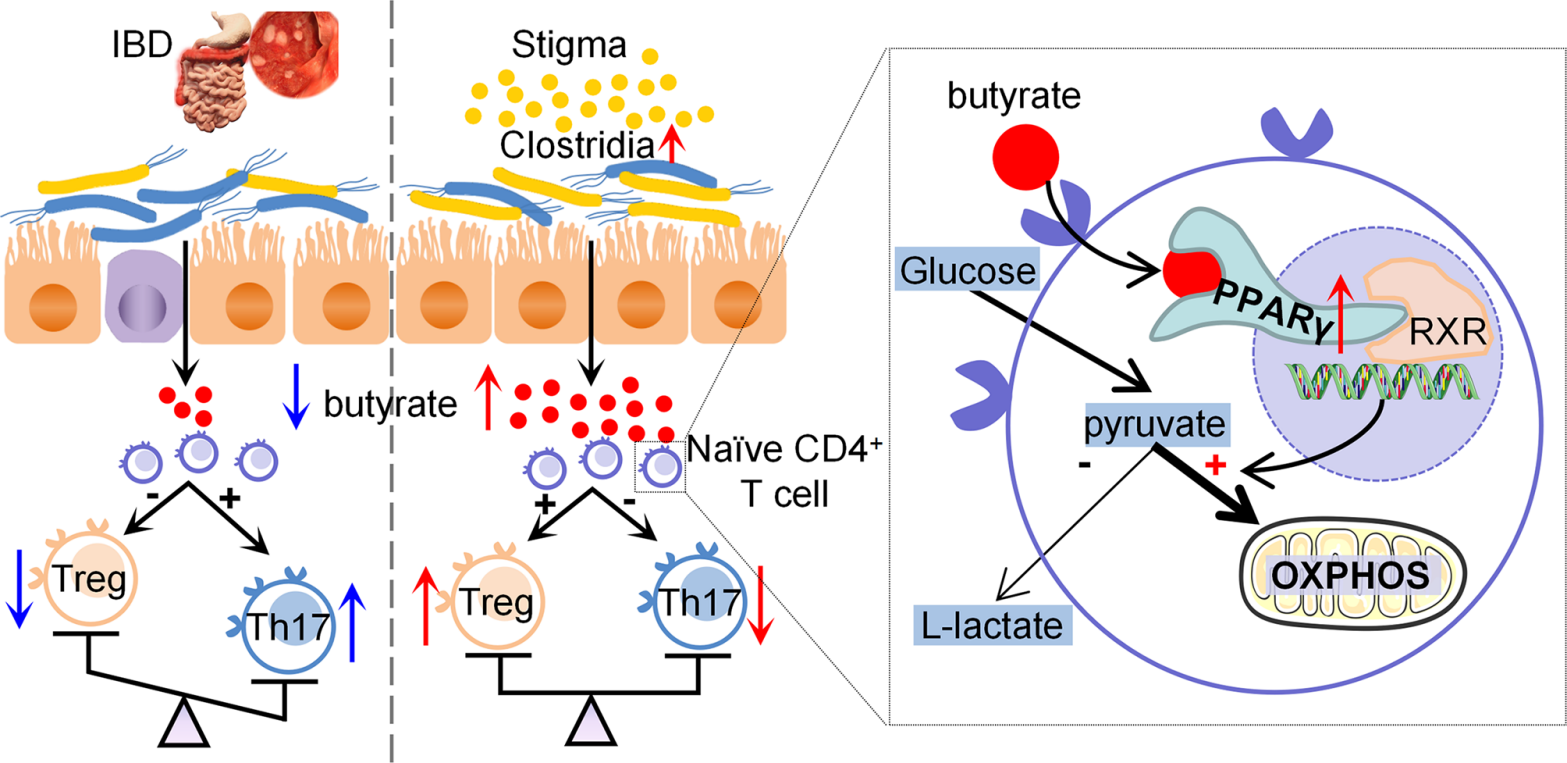
Diagram of the potential mechanisms involved in therapeutic effects of stigmasterol on colitis.

IBD is increasingly becoming an important global public health issue (26). Thus, a more thorough understanding of the pathogenesis of and optimal treatment strategies for IBD is needed. Recently, phytosterols, which are highly prevalent in the human diet, have received increasing attention because of their antioxidant and anti-inflammatory features (27). Phytosterols have potential benefits in treating gastrointestinal inflammatory diseases. The present study found that stigmasterol restores the Treg/Th17 balance by increasing butyrate-producing bacteria, attenuating DSS-induced colitis. Additionally, butyrate modulates the differentiation of Treg and Th17 cells through PPARγ, and the specific mechanism is linked to T cell metabolism. Furthermore, the gut microbiota plays a significant role in the treatment of colitis by stigmasterol (**Figure 9**).

The Treg/Th17 balance is essential to maintain gut homeostasis (8). When microbes and pathogens invade the intestinal mucosal barrier, inflammatory cytokines are produced by activated APCs and may promote differentiation into Th17 cells, generating a protective immune response necessary for pathogen clearance (28). In pathological situations, the aberrant Th17 response and IL-17A production in great excess of the immune tolerance of Tregs induce severe tissue damage and result in IBD (29).

Gut microbiota antigens are essential for T cell differentiation and activation (30, 31). The diversity of the intestinal flora in IBD patients is reduced, and this effect manifests as disruption of the beneficial microflora and increased colonization of pathogenic bacteria (32, 33). Colonization of germ-free (GF) mice with the flora of IBD patients can markedly increase Th17 cells and decrease Treg cells (12). Here, we demonstrated for the first time that stigmasterol significantly restores the abundance and diversity of the gut microbiota in the context of colitis. Stigmasterol increases the composition of *Ruminococcus*, *Prevotella* and *Helicobacter*. Previous studies have demonstrated that an increased level of *Ruminococcus* is closely associated with Treg cell differentiation (34). *Prevotella* generates anti-inflammatory metabolites, promotes the differentiation of Treg/Tr1 cells and reduces Th17 polarization in the gut (35). *Helicobacter* colonization regulates the Treg/Th17 cell balance and induces the polarization of macrophages towards the anti-inflammatory M2 phenotype, preventing colitis (36).

In addition to gut flora, SCFAs, the metabolic products of bacterial dietary fibre, affects Treg and Th17 cell differentiation (15, 37). Our studies demonstrated that stigmasterol increases the abundance of Clostridia. Clostridia is the primary producer of SCFAs, which are a significant energy source in colonocytes and are critical to maintain gut health (38). *Odoribacter (* 39), *Prevotella (* 40), *Oscillospira (* 41), *Paraprevotella* and *Ruminococcu*s *(* 42), the abundance of which is elevated by stigmasterol, also synthetize SCFAs, particularly butyrate. Simultaneously, stigmasterol increased the expression levels of SCFAs, comprising acetate, propionate, and butyrate, in faeces. Correlation analysis has suggested that the butyrate level is most significantly associated with the abundance of components of the intestinal flora. Previous studies have demonstrated that butyrate synthesis was reduced in IBD patients. However, direct butyrate administration may be therapeutically inadequate. Compared to directly supplementing butyrate, regulating the intestinal flora may provide a more consistent and effective changes in intestinal butyrate concentration (43). In accordance with the present results, previous studies have demonstrated that butyrate promotes Treg cell differentiation and suppresses Th17 cell differentiation (44).

Next, we aimed to explore the mechanism by which butyrate affects the Treg/Th17 balance. Previous studies have indicated an important role for PPARγ in butyrate effects. Butyrate protects mice against colitis and increases the PPARγ expression levels *in vivo (* 45, 46). Butyrate activates PPARγ signalling, contributing to the maintenance of intestinal homeostasis (47, 48). PPARγ is a ligand-activated transcription factor of mitochondrial gene expression that is primarily expressed in adipose tissue and the immune system (49), plays critical roles in lipid and glucose metabolism, and mediates anti-inflammatory activity (50). PPARγ is closely related to immunity in the body and is a potential curative target for IBD (51). Previous research has shown the relevance of PPARγ and Treg/Th17 balance in experimental animal model (52). PPARγ contributes significantly to the proliferation and function of Treg cells in adipose tissue (53) and mediates the transformation of Th17 cells into Treg cells (54). However, the relationship among butyrate, PPARγ and T cell differentiation has not been fully elucidated. Herein, we demonstrated that butyrate regulates Treg and Th17 cell differentiation *in vitro* by activating PPARγ.

PPARγ is a key adjustor of metabolism, and T-cell energy metabolism is tightly linked to the proliferation and differentiation of Treg and Th17 cells (55). Therefore, we performed further studies to explore whether butyrate affects cell metabolism by activating PPARγ. PPARγ is regulated by glucose availability and the mammalian target of rapamycin (mTOR) signalling pathway (56).Recently, signalling pathways such as the mTOR and HIF-1α pathways were found to regulate Treg and Th17 cell differentiation and immunosuppression by modulating cellular energy metabolism (57). HIF-1α expression is upregulated in inflammatory tissues (58), and HIF-1α participates in the pathological progression of IBD. HIF1α-dependent transcriptional programming is critical for mediating glycolytic activity (59). mTORC1, the master regulator of cellular metabolism, enhances glycolysis by upregulating HIF-1α expression.

HIF-1α directly activates RORγt and enhances TH17 cell differentiation but suppresses Treg cell differentiation by binding the Foxp3 protein and targeting it for degradation (60). Glycolytic activity is increased under Th17-polarizing conditions. When mTORC1 activity is inhibited, HIF-1α expression is downregulated, and PPARγ expression is upregulated, resulting in blockade of glycolysis, thereby suppressing Th17 cell differentiation and promoting Foxp3+ Treg cell differentiation, which reduces the inflammatory response (61, 62). Treg cells and Th17 cells exhibit opposite cellular metabolic signatures. Treg cells rely on glycolysis, fatty acid oxidation (FAO) and OXPHOS (63). The main source of energy for Treg cells is OXPHOS, whereas Th17 cells mainly depend on glycolysis and glutaminolysis to maintain their proliferation, differentiation and function (64). Decreased PPARγ expression influences mitochondrial bioenergetics, such as reducing the OCR and promoting glycolysis. In our study, we found that butyrate, which was produced by gut flora, shifted energy metabolism from glycolysis towards OXPHOS through PPARγ activation and promoted Treg cell differentiation.

Interestingly, when the intestinal microbiota was consumed, the generation of butyrate was reduced, and the protective effects of stigmasterol against DSS-induced colitis were abolished. This finding illustrates not only that stigmasterol affects the intestinal flora composition but also that the flora alters the therapeutic effect of stigmasterol against IBD. The drug and gut flora interact affecting the composition of the intestinal flora (65, 66); additionally, the drug can be metabolically inactivated or transformed by the flora to exert its effect or produce side effects (67–69). Microbes interact with the host immune system, directly or indirectly influencing the pharmaceutical activities of immunomodulatory drugs and playing crucial roles in drug treatment (70). Hence, the gut microbiota can affect an individual’s response to drugs.

Stigmasterol must be obtained through the diet and absorbed through the intestines, because it cannot be biosynthesized by the body. The absorption rate of phytosterols in the body is low. The gut flora plays a pivotal role in the therapeutic response to drugs with low solubility and permeability that remain in the gastrointestinal tract for an extended period of time and are biotransformed by flora. Phytosterols can regulate the metabolism of the intestinal flora. When the intake of phytosterols is sufficient, the gut microbiota can preferentially use phytosterols (71).

Previous studies have demonstrated that stigmasterol has a protective effect on gastric and duodenal ulcers, including ulcers associated with nonsteroidal anti-inflammatory drugs (NSAIDs) (72). Additionally, stigmasterol alleviates cutaneous allergic responses (73) and promotes the healing of ulcers (74). Furthermore, stigmasterol prevents hypoxia-reoxygenation injury by modulating mitophagy (75). It has a protective effect in acute and chronic pain models and can relieve mechanical allodynia caused by surgical incisions and partial ligation of the sciatic nerve (76). This finding suggests that stigmasterol is a potential option in healthy individuals or in traumatic conditions to protect gut mucosa. Our findings contribute to the understanding of the mechanisms by which stigmasterol exerts a protective effect against IBD by modulating the levels of microbiota-derived SCFAs, especially butyrate.

Taken together, our study revealed that stigmasterol decreases colonic inflammation in a gut flora-dependent manner. The increase in the content of gut microbiota-derived butyrate by stigmasterol leads to activation of PPARγ to promote changes in T cell metabolism from glycolysis to OXPHOS. Butyrate restores the Treg/Th17 balance in patients with IBD through metabolic reprogramming. PPARγ is a vital target that increases the differentiation of naïve CD4+ T cells into Treg cells instead of Th17 cells. In general, our results demonstrate the critical role of stigmasterol as a potential modulator of the intestinal flora in the prevention and treatment of IBD. These findings improve understanding of the molecular mechanisms of IBD and provide new potential treatment strategies for IBD, but the association between IBD and the gut flora remains to be further explored.

## Data Availability Statement

The original contributions presented in the study are included in the article/**Supplementary Material**. Further inquiries can be directed to the corresponding authors.

## Ethics Statement

The studies involving human participants were reviewed and approved by the Medical Ethics Committee of the Academic Medical Center of Guangzhou University of Chinese Medicine. The patients/participants provided their written informed consent to participate in this study. The animal study was reviewed and approved by the Institutional Animal Ethics Committee of the First Affiliated Hospital of Guangzhou University of Chinese Medicine. Written informed consent was obtained from the individual(s) for the publication of any potentially identifiable images or data included in this article.

## Author Contributions

FL, STW, and LH designed the study. STW, LH, and ZZ conducted the experiments. ZZ, RZ, and SHW collected clinical data and blood samples. LH, STW, and HM analysed the data and performed the statistical analyses. STW wrote the manuscript and FL critically revised the article for essential intellectual content. All authors contributed to the article and approved the submitted version.

## Funding

This work is supported by the Natural Science Foundation of Guangdong Province (No. 2018A030310614), Guangzhou Science, Technology and Innovation Commission (No. 201904010234), Major projects of first-class disciplines in Guangzhou University of TCM (No. 2021XK18), Project of Department of Education of Guangdong Province (No. 2017KQNCX045), and the National Natural Science Foundation of China (81903963).

## Conflict of Interest

The authors declare that the research was conducted in the absence of any commercial or financial relationships that could be construed as a potential conflict of interest.

## Publisher’s Note

All claims expressed in this article are solely those of the authors and do not necessarily represent those of their affiliated organizations, or those of the publisher, the editors and the reviewers. Any product that may be evaluated in this article, or claim that may be made by its manufacturer, is not guaranteed or endorsed by the publisher.

## Supplementary Material

Supplementary Figure 1 PCA, PCoA, PLS-DA and NMDS analysis of the intestinal microbiota in the water, DSS and stigma + DSS groups (n = 5 per group).

Supplementary Figure 2 Histogram of linear discriminant analysis (LDA) scores. Blue represents microbial taxa with significant effects in the DSS group, and red represents microbial taxa with significant effects in the stigma + DSS group. The threshold for discriminative features was a logarithmic LDA score > 3 (n = 5 per group).

Supplementary Figure 3 Therapeutic FMT mitigates DSS-induced colitis. **(A)** Schematic of FMT studies involving stigmasterol. First, WT mice were pretreated for 5 days with ABX to deplete the gut microbiota. Next, faecal microbiota was intragastrically administered for 5 days, after which the mice were subjected to 3 days of intestinal rest. Next, the mice were administered 2.5% DSS for 7 days followed by normal water for an additional 3 days to induce acute colitis. **(B, C)** Body weight **(B)** and DAI score **(C)** of the FMT (DSS) (blue dotted line) and FMT (stigma + DSS) groups (red dotted line) (n = 10 per group). **(D)** Macroscopic appearance and length of the colon (n = 10 per group). **(E)** Representative microscopy images (40× and 200× magnification) of H&E-stained colon tissues and corresponding histological scores (n = 10 per group). **(F)** Analysis of inflammatory cytokine expression in distal colonic tissues. IL-6, IL-1β and TNF-α cytokine levels in colon tissue homogenates from the FMT (DSS) and FMT (stigma + DSS) groups were measured by ELISA (n = 5 per group). **(G)** Survival curves of the FMT (DSS) group and FMT (stigma + DSS) groups (n = 10 per group). **(B-G)** The data are representative of three independent experiments. * P < 0.05, ** P < 0.01, *** P < 0.001.

Supplementary Figure 4 Therapeutic FMT restores the balance between Tregs and Th17 cells. **(A)** The number of Treg cells in the spleen and MLNs of the FMT (DSS) and FMT (stigma + DSS) groups was analysed by flow cytometry. **(B, C)** Bar charts showing the percentage of CD4^+^Foxp3^+^ (Treg) cells among the CD4^+^ T cell subsets in the spleen **(B)** and MLNs **(C). (D)** The number of Th17 cells in the spleen and MLNs of the FMT (DSS) and FMT (stigma + DSS) groups was analysed by flow cytometry. **(E, F)** Bar charts showing the percentage of CD4^+^IL17A^+^(Th17) cells in the spleen **(E)** and MLNs **(F). (A-F)** n = 3 mice per group. **(G)** ELISA of serum IL-10, TGF-β and IL-17A levels. n = 5 mice per group. **(A-G)** Data are representative of three independent experiments. * P < 0.05, ** P < 0.01.

Supplementary Figure 5 Correlation between the relative abundances of Clostridia in the gut and butyrate concentrations in the faeces of the water group, DSS group and stigma + DSS group.

Supplementary Figure 6 Immunofluorescence staining revealed that the expression of PPARγ in colonic tissue was reduced in DSS colitis. However, compared with the DSS group, stigmasterol treatment increased the PPARγ expression. Red represented PPARγ, and blue represented DAPI. The magnification is 400×.

## Abbreviations

IBD, inflammatory bowel disease; UC, ulcerative colitis; CD, Crohn’s disease; DSS, dextran sodium sulfate; DAI, disease activity index; SCFAs, short-chain fatty acids; BA, butyric acid; PPARγ, peroxisome proliferator-activated receptor gamma; 5-ASA, 5-aminosalicylic acid; IL-6, interleukin-6; IL-1β, interleukin-1β; TNF-α, tumour necrosis factor-α; Treg, regulatory T; Th17, T helper type 17; FMT, faecal microbiota transplantation; GD WT, gut microbiota-depleted wild-type; OTU, operational taxonomic units; PCA, principal component analysis; PCoA, principal coordinate analysis; PLS-DA, partial least squares–discriminant analyses; NMDS, nonmetric multidimensional scaling; ELISA, enzyme-linked immunosorbent assay; OCR, oxygen consumption rate; OXPHOS, oxidative phosphorylation; glycoPER, glycolytic proton efflux rate.
