# Supplementary figures and images for: Stigmasterol Restores the Balance of Treg/Th17 Cells by Activating the Butyrate-PPARγ Axis in Colitis
Source: Front Immunol. 2021 Oct 6;12:741934. doi: 10.3389/fimmu.2021.741934 (PMC8526899; doi:10.3389/fimmu.2021.741934)

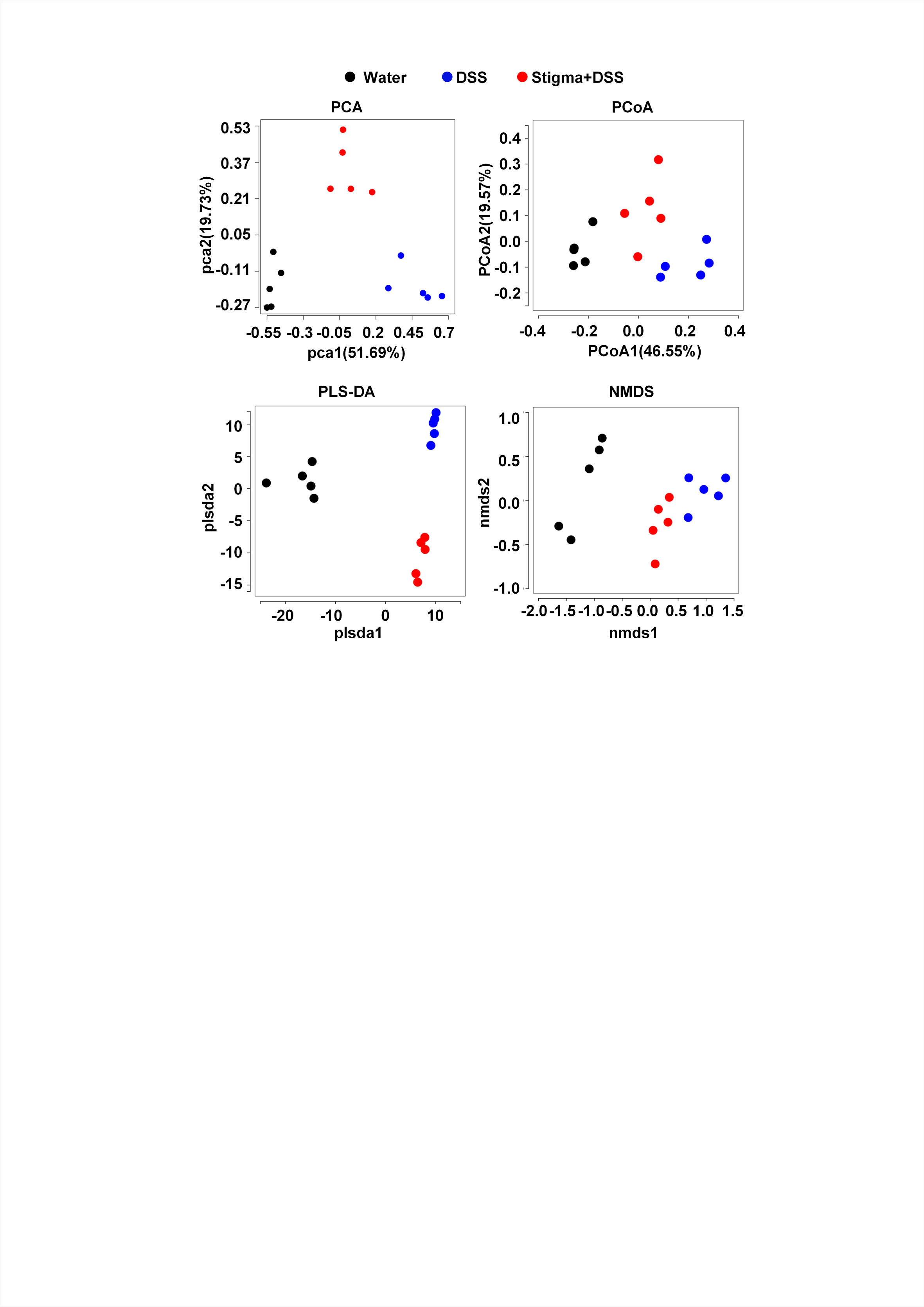

Supplement: Supplementary Figure 1 — PCA, PCoA, PLS-DA and NMDS analysis of the intestinal microbiota in the water, DSS and stigma + DSS groups (n = 5 per group). [file Image_1.tif]

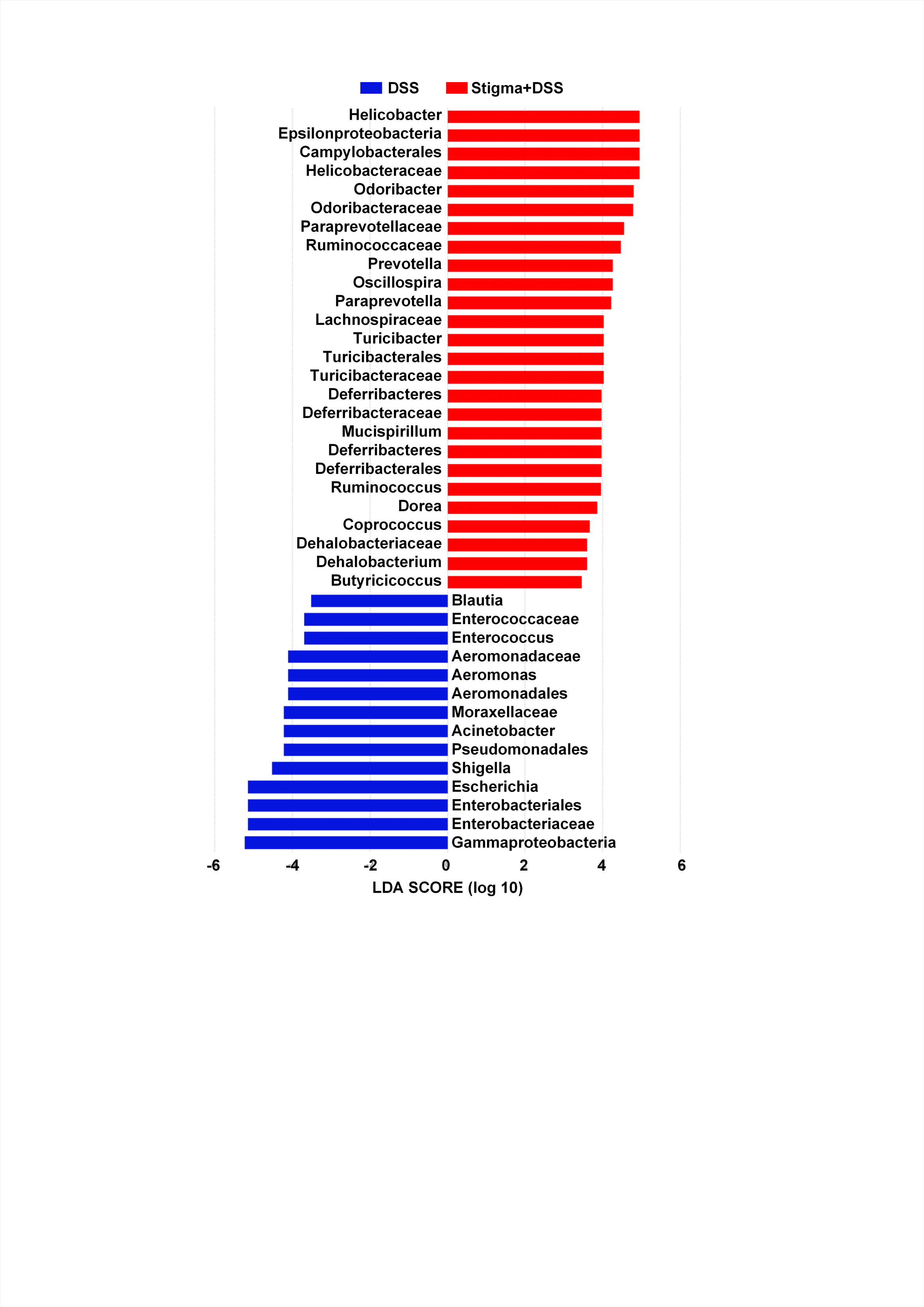

Supplement: Supplementary Figure 2 — Histogram of linear discriminant analysis (LDA) scores. Blue represents microbial taxa with significant effects in the DSS group, and red represents microbial taxa with significant effects in the stigma + DSS group. The threshold for discriminative features was a logarithmic LDA score > 3 (n = 5 per group). [file Image_2.tif]

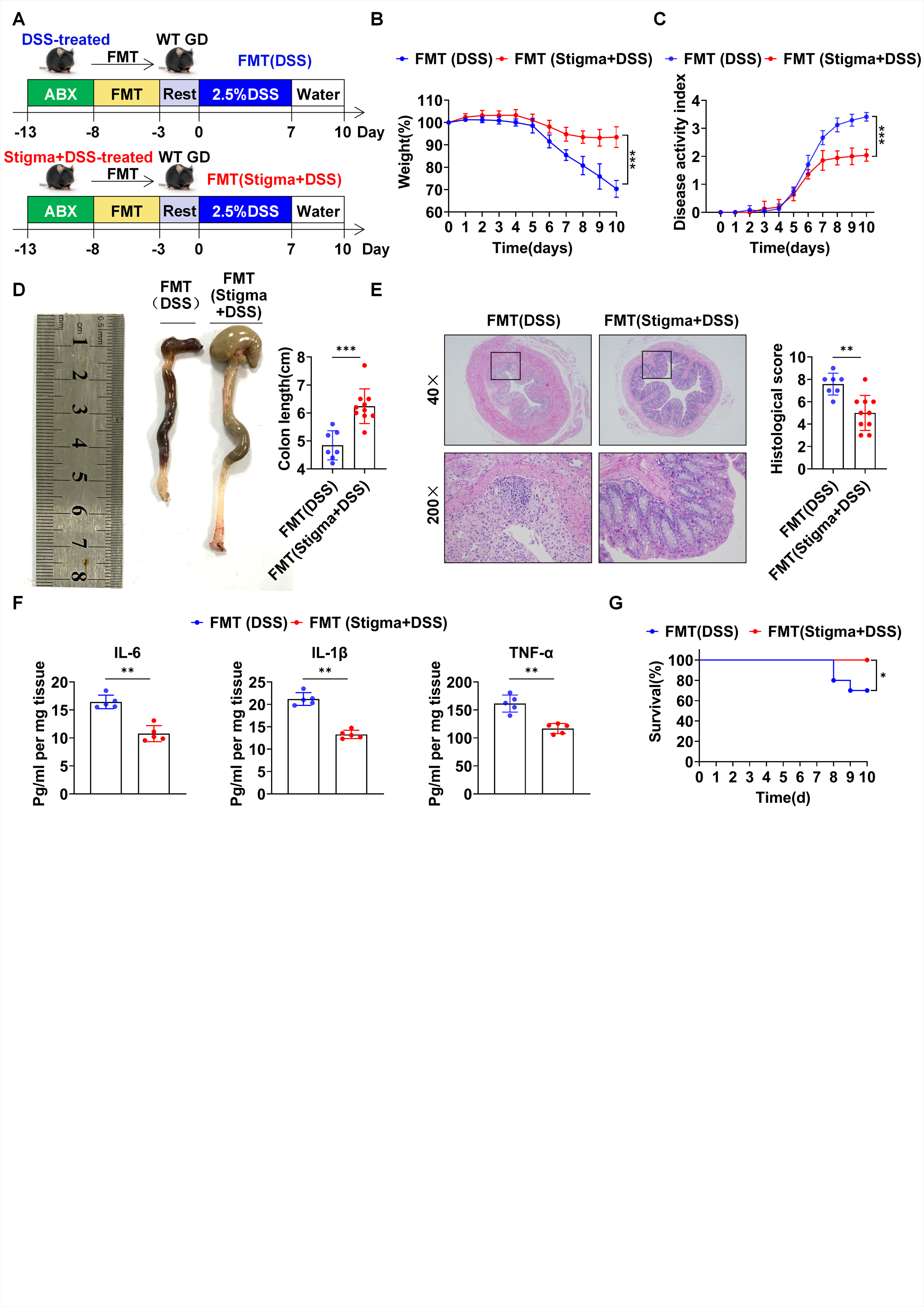

Supplement: Supplementary Figure 3 — Therapeutic FMT mitigates DSS-induced colitis. (A) Schematic of FMT studies involving stigmasterol. First, WT mice were pretreated for 5 days with ABX to deplete the gut microbiota. Next, faecal microbiota was intragastrically administered for 5 days, after which the mice were subjected to 3 days of intestinal rest. Next, the mice were administered 2.5% DSS for 7 days followed by normal water for an additional 3 days to induce acute colitis. (B, C) Body weight (B) and DAI score (C) of the FMT (DSS) (blue dotted line) and FMT (stigma + DSS) groups (red dotted line) (n = 10 per group). (D) Macroscopic appearance and length of the colon (n = 10 per group). (E) Representative microscopy images (40× and 200× magnification) of H&E-stained colon tissues and corresponding histological scores (n = 10 per group). (F) Analysis of inflammatory cytokine expression in distal colonic tissues. IL-6, IL-1β and TNF-α cytokine levels in colon tissue homogenates from the FMT (DSS) and FMT (stigma + DSS) groups were measured by ELISA (n = 5 per group). (G) Survival curves of the FMT (DSS) group and FMT (stigma + DSS) groups (n = 10 per group). (B-G) The data are representative of three independent experiments. * P < 0.05, ** P < 0.01, *** P < 0.001. [file Image_3.tif]

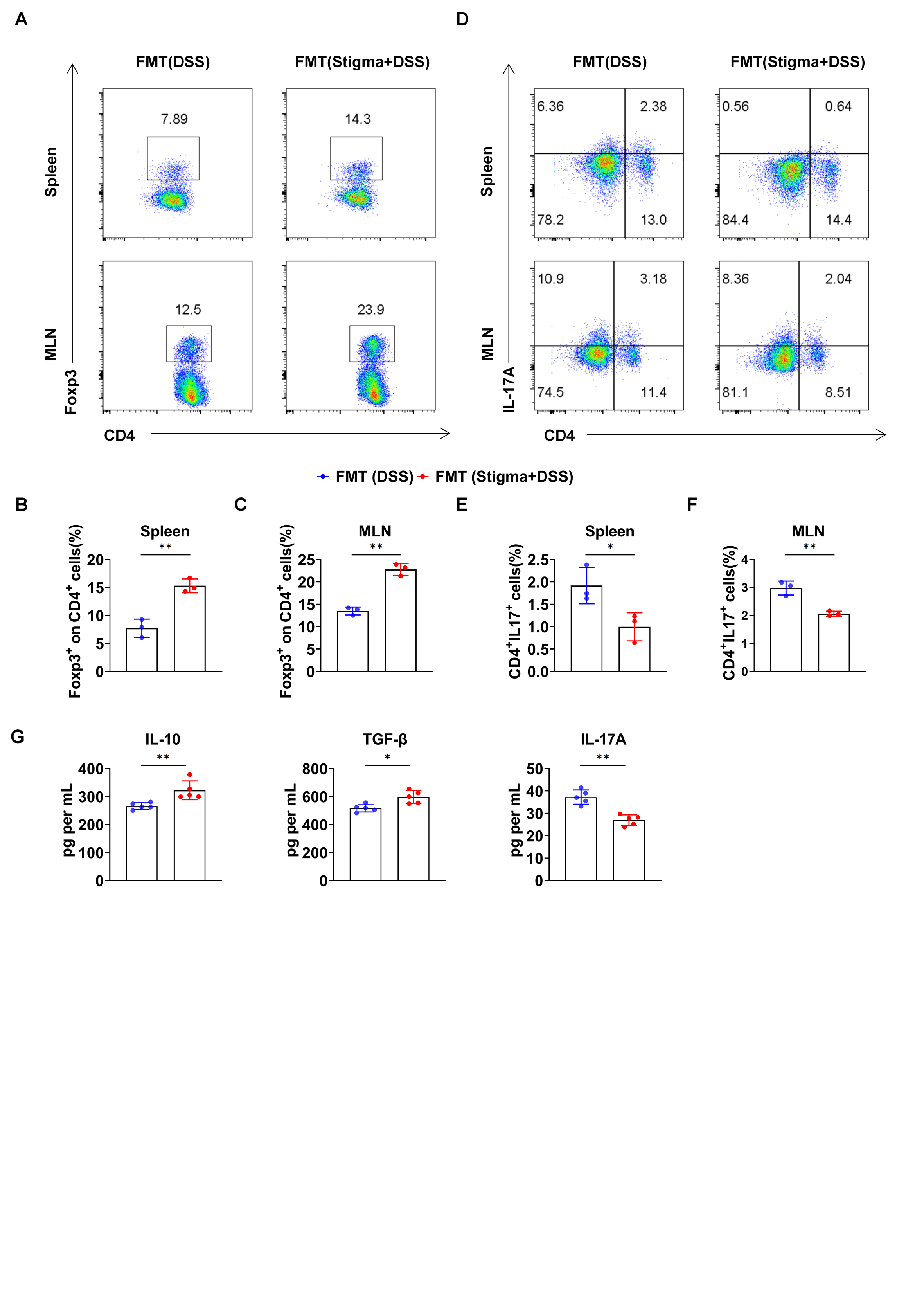

Supplement: Supplementary Figure 4 — Therapeutic FMT restores the balance between Tregs and Th17 cells. (A) The number of Treg cells in the spleen and MLNs of the FMT (DSS) and FMT (stigma + DSS) groups was analysed by flow cytometry. (B, C) Bar charts showing the percentage of CD4+Foxp3+ (Treg) cells among the CD4+ T cell subsets in the spleen (B) and MLNs (C). (D) The number of Th17 cells in the spleen and MLNs of the FMT (DSS) and FMT (stigma + DSS) groups was analysed by flow cytometry. (E, F) Bar charts showing the percentage of CD4+IL17A+(Th17) cells in the spleen (E) and MLNs (F). (A-F) n = 3 mice per group. (G) ELISA of serum IL-10, TGF-β and IL-17A levels. n = 5 mice per group. (A-G) Data are representative of three independent experiments. * P < 0.05, ** P < 0.01. [file Image_4.tif]

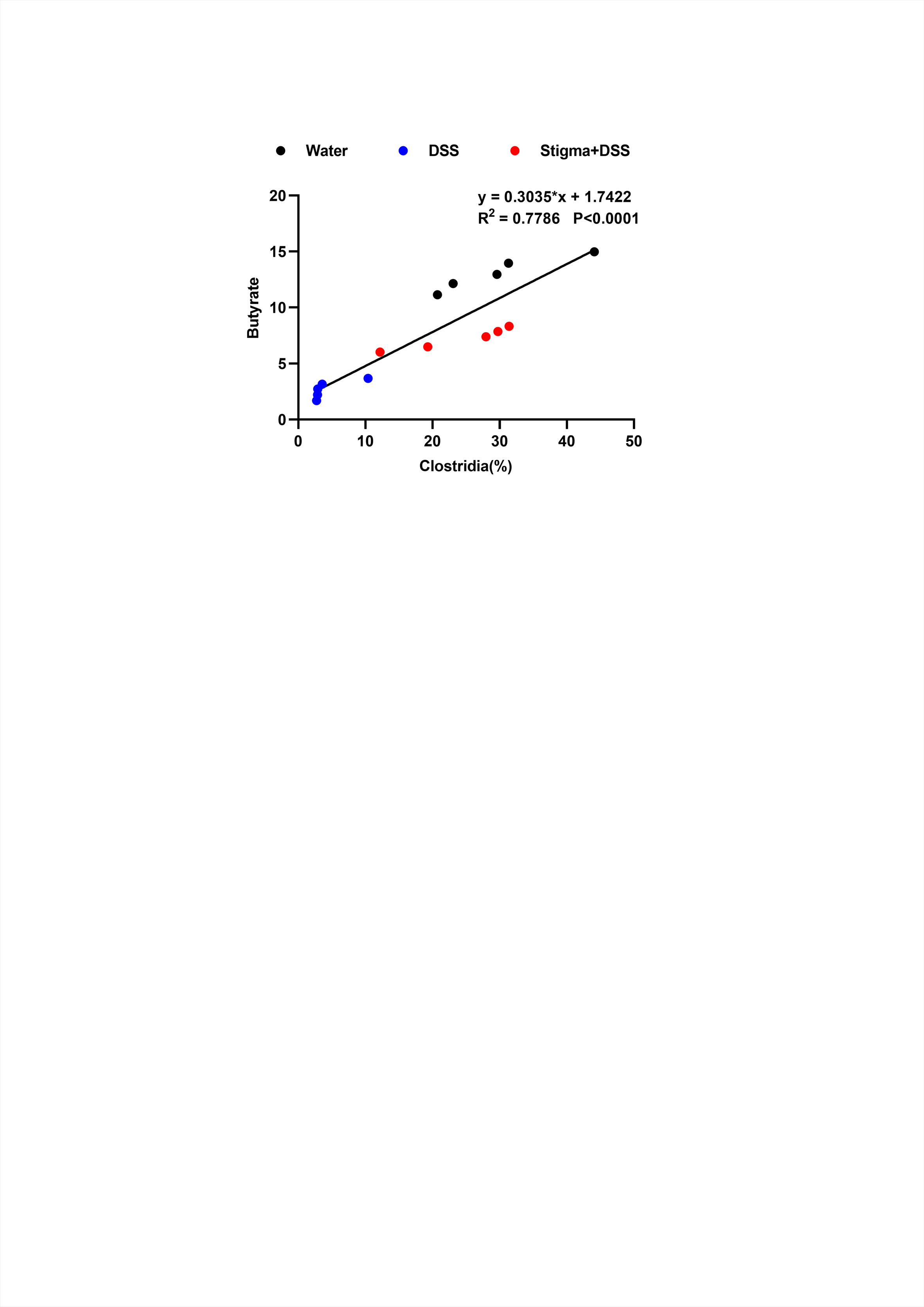

Supplement: Supplementary Figure 5 — Correlation between the relative abundances of Clostridia in the gut and butyrate concentrations in the faeces of the water group, DSS group and stigma + DSS group. [file Image_5.tif]

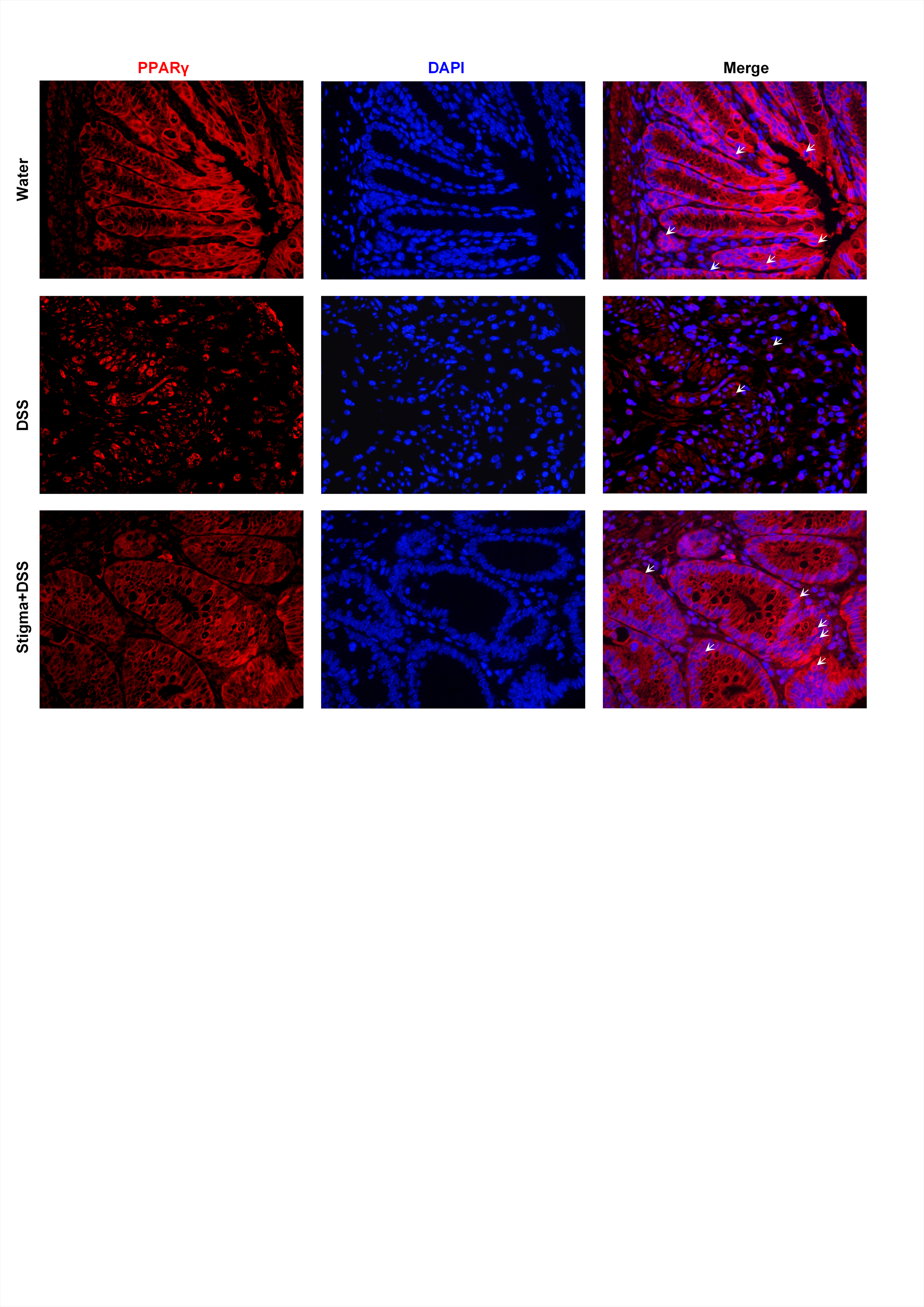

Supplement: Supplementary Figure 6 — Immunofluorescence staining revealed that the expression of PPARγ in colonic tissue was reduced in DSS colitis. However, compared with the DSS group, stigmasterol treatment increased the PPARγ expression. Red represented PPARγ, and blue represented DAPI. The magnification is 400×. [file Image_6.tif]
