# Supplementary material for: Stigmasterol Restores the Balance of Treg/Th17 Cells by Activating the Butyrate-PPARγ Axis in Colitis
Source: Front Immunol. 2021 Oct 6;12:741934. doi: 10.3389/fimmu.2021.741934 (PMC8526899; doi:10.3389/fimmu.2021.741934)
Supplement: Supplementary file 7 [file DataSheet_1.doc]

Supplementary Table S1. Scoring criteria of disease activity index (DAI)

| Score | Weight loss | Stool consistency | Blood stool |
| --- | --- | --- | --- |
| 0 | no loss | normal | no blood |
| 1 | 1-5% | loose stool |  |
| 2 | 5-10% | watery diarrhea | presence of blood |
| 3 | 10-20% | slimy diarrhea, little blood |  |
| 4 | ＞20% | severe watery diarrhea with blood | gross bleeding |

Supplementary Table S2. Scoring criteria of histopathology

| Score | Crypt architecture | degree  of inflammatory cell infiltration | mucosa thickening | crypt abscess | goblet cell depletion |
| --- | --- | --- | --- | --- | --- |
| 0 | normal | normal | base of crypt sits on the muscularis mucosae | absent | absent |
| 1 | mild | mild | mild | present | present |
| 2 | moderate | moderate | moderate | - | - |
| 3 | severe crypt distortion with loss of entire crypts | dense inflammatory infiltrate | marked muscle  thickening | - | - |

Supplementary Table S3. Differences in microbial abundance at the genus level in fecal samples.

|  | Water | DSS | Stigma+DSS | P-value  (Water versus DSS) | P-value  (DSS versus Stigma+DSS) |
| --- | --- | --- | --- | --- | --- |
| Acetatifactor | 0.290±0.124 | 0.010±0.004 | 0.314±0.434 | 0.0007 | 0.1989 |
| Acinetobacter | 0.003±0.005 | 0.015±0.012 | 0.001±0.002 | 0.1467 | 0.0509 |
| Aeromonas | 0.000±0.000 | 0.140±0.166 | 0.000±0.001 | 0.1753 | 0.1313 |
| Aestuariispira | 0.016±0.021 | 0.000±0.001 | 0.002±0.004 | 0.1809 | 0.4533 |
| Akkermansia | 2.331±1.638 | 0.028±0.018 | 0.247±0.411 | 0.0125 | 0.3175 |
| Alistipes | 0.499±0.417 | 1.410±0.507 | 2.063±0.847 | 0.0140 | 0.2227 |
| Allobaculum | 0.551±0.286 | 1.252±0.914 | 0.294±0.136 | 0.2529 | 0.0421 |
| Alloprevotella | 0.149±0.182 | 0.000±0.000 | 0.004±0.006 | 0.1101 | 0.2364 |
| Anaerofustis | 0.000±0.001 | 0.000±0.001 | 0.000±0.000 | 0.8726 | 0.3466 |
| Anaeroplasma | 0.241±0.382 | 0.002±0.003 | 0.012±0.014 | 0.1985 | 0.1957 |
| Anaerotruncus | 0.000±0.000 | 0.000±0.000 | 0.001±0.001 | - | 0.0402 |
| Anaerovorax | 0.002±0.003 | 0.005±0.003 | 0.004±0.004 | 0.4824 | 0.7078 |
| Bacteroides | 0.951±1.056 | 30.388±7.303 | 19.147±5.581 | 0.0000 | 0.0402 |
| Barnesiella | 2.929±2.139 | 0.044±0.068 | 0.016±0.011 | 0.0152 | 0.4348 |
| Bifidobacterium | 0.328±0.384 | 0.022±0.023 | 0.016±0.015 | 0.1109 | 0.6541 |
| Blautia | 0.017±0.021 | 0.496±0.301 | 0.023±0.009 | 0.0341 | 0.0136 |
| Butyricicoccus | 0.126±0.051 | 0.003±0.003 | 0.308±0.244 | 0.1581 | 0.0369 |
| Butyricimonas | 0.028±0.018 | 0.738±0.297 | 0.207±0.109 | 0.0059 | 0.0100 |
| Butyrivibrio | 0.083±0.110 | 0.005±0.006 | 0.034±0.020 | 0.1671 | 0.0230 |
| Catabacter | 0.002±0.001 | 0.002±0.002 | 0.000±0.000 | 0.8193 | 0.2079 |
| Christensenella | 0.000±0.000 | 0.000±0.001 | 0.000±0.000 | 0.3893 | 0.3466 |
| Clostridium_III | 0.005±0.001 | 0.001±0.001 | 0.000±0.001 | 0.0002 | 0.5397 |
| Clostridium_IV | 0.645±0.117 | 0.121±0.111 | 0.358±0.112 | 0.0001 | 0.0169 |
| Clostridium_XI | 0.000±0.000 | 0.337±0.329 | 0.009±0.014 | 0.1038 | 0.0813 |
| Clostridium_XVIII | 0.012±0.018 | 0.288±0.060 | 0.378±0.449 | 0.0000 | 0.7008 |
| Clostridium_XlVa | 13.161±5.485 | 0.659±0.461 | 6.819±3.352 | 0.0011 | 0.0066 |
| Clostridium_XlVb | 0.120±0.120 | 0.114±0.049 | 0.523±0.419 | 0.5104 | 0.0892 |
| Clostridium_sensu_stricto | 0.003±0.004 | 0.741±0.638 | 0.186±0.099 | 0.0617 | 0.1238 |
| Coprobacillus | 0.002±0.001 | 0.000±0.000 | 0.001±0.001 | 0.0438 | 0.3466 |
| Coprococcus | 0.001±0.001 | 0.001±0.002 | 0.006±0.003 | 0.4205 | 0.0441 |
| Corynebacterium | 0.000±0.000 | 0.000±0.000 | 0.000±0.001 | - | 0.3466 |
| Desulfovibrio | 1.033±0.368 | 0.846±0.437 | 4.072±2.329 | 0.5153 | 0.0261 |
| Dorea | 0.001±0.001 | 0.000±0.000 | 0.000±0.001 | 0.2967 | 0.3466 |
| Eisenbergiella | 0.000±0.000 | 0.001±0.002 | 0.013±0.016 | 0.1867 | 0.1882 |
| Empedobacter | 0.000±0.000 | 0.002±0.003 | 0.000±0.000 | 0.3893 | 0.3466 |
| Enterococcus | 0.000±0.001 | 0.238±0.160 | 0.001±0.001 | 0.0435 | 0.0178 |
| Enterorhabdus | 0.004±0.005 | 0.002±0.002 | 0.005±0.009 | 0.3041 | 0.4570 |
| Escherichia | 0.006±0.008 | 25.028±14.782 | 0.340±0.264 | 0.0299 | 0.0102 |
| Eubacterium | 0.182±0.149 | 0.324±0.098 | 0.042±0.028 | 0.3483 | 0.0006 |
| Faecalicoccus | 0.000±0.000 | 0.022±0.030 | 0.001±0.001 | 0.2192 | 0.1938 |
| Flavonifractor | 0.511±0.479 | 0.283±0.428 | 0.449±0.317 | 0.4020 | 0.5515 |
| Gemella | 0.000±0.000 | 0.001±0.001 | 0.000±0.001 | 0.2139 | 0.4231 |
| Haemophilus | 0.000±0.001 | 0.001±0.003 | 0.000±0.000 | 0.5777 | 0.3466 |
| Helicobacter | 0.485±0.244 | 2.229±1.285 | 13.238±8.725 | 0.1266 | 0.0371 |
| Intestinimonas | 0.149±0.155 | 0.008±0.006 | 0.123±0.124 | 0.1057 | 0.1035 |
| Klebsiella | 0.000±0.000 | 0.937±1.161 | 0.001±0.001 | 0.1906 | 0.1454 |
| Lachnospiracea_incertae_sedis | 0.000±0.000 | 0.001±0.001 | 0.004±0.005 | 0.1083 | 0.2515 |
| Lactobacillus | 2.127±2.612 | 0.339±0.565 | 0.226±0.123 | 0.1668 | 0.7050 |
| Macrococcus | 0.000±0.000 | 0.000±0.001 | 0.000±0.000 | 0.3893 | 0.3466 |
| Megamonas | 0.000±0.001 | 0.000±0.000 | 0.000±0.000 | 0.2967 | - |
| Mucispirillum | 0.017±0.009 | 0.255±0.295 | 1.314±1.470 | 0.0862 | 0.1956 |
| Odoribacter | 0.292±0.519 | 1.434±1.117 | 8.645±3.800 | 0.0600 | 0.0497 |
| Olsenella | 0.017±0.011 | 0.069±0.067 | 0.024±0.008 | 0.1684 | 0.2233 |
| Oscillibacter | 0.449±0.218 | 0.071±0.070 | 0.667±0.810 | 0.0054 | 0.1809 |
| Papillibacter | 0.024±0.031 | 0.004±0.007 | 0.004±0.006 | 0.1814 | 0.9252 |
| Parabacteroides | 0.156±0.092 | 3.808±2.253 | 1.252±0.635 | 0.0175 | 0.0605 |
| Paraprevotella | 0.794±0.430 | 0.450±0.849 | 2.742±0.963 | 0.7006 | 0.0064 |
| Parasutterella | 0.413±0.364 | 0.467±0.498 | 0.278±0.206 | 0.7766 | 0.5040 |
| Parvibacter | 0.011±0.009 | 0.038±0.028 | 0.006±0.004 | 0.2021 | 0.0565 |
| Pasteurella | 0.002±0.001 | 5.593±8.622 | 0.019±0.010 | 0.2761 | 0.2321 |
| Prevotella | 1.928±1.702 | 0.014±0.019 | 0.466±0.292 | 0.0411 | 0.0150 |
| Proteus | 0.000±0.000 | 0.019±0.038 | 0.000±0.000 | 0.3893 | 0.3466 |
| Pseudoflavonifractor | 0.163±0.064 | 0.032±0.020 | 0.187±0.169 | 0.0019 | 0.1057 |
| Pseudomonas | 0.000±0.001 | 0.000±0.000 | 0.000±0.000 | 0.2967 | - |
| Rikenella | 0.120±0.143 | 0.000±0.001 | 0.002±0.002 | 0.0956 | 0.3507 |
| Romboutsia | 0.001±0.001 | 0.075±0.046 | 0.473±0.496 | 0.0058 | 0.1492 |
| Roseburia | 0.001±0.001 | 0.001±0.001 | 0.000±0.000 | 0.7639 | 0.1413 |
| Ruminococcus | 0.901±0.879 | 0.013±0.017 | 0.310±0.032 | 0.0055 | 0.0387 |
| Ruminococcus2 | 0.000±0.000 | 0.001±0.001 | 0.010±0.010 | 0.2951 | 0.1304 |
| Saccharibacteria | 0.052±0.030 | 0.002±0.003 | 0.014±0.016 | 0.0047 | 0.1844 |
| Senegalimassilia | 0.000±0.001 | 0.000±0.001 | 0.002±0.001 | 0.8798 | 0.0654 |
| Sporobacter | 0.119±0.054 | 0.023±0.034 | 0.228±0.282 | 0.0083 | 0.1877 |
| Staphylococcus | 0.000±0.001 | 0.004±0.006 | 0.000±0.001 | 0.3397 | 0.2787 |
| Stenotrophomonas | 0.005±0.011 | 0.001±0.002 | 0.000±0.001 | 0.3914 | 0.6704 |
| Streptococcus | 0.002±0.002 | 4.971±3.792 | 0.426±0.423 | 0.2834 | 0.0478 |
| Subdoligranulum | 0.000±0.001 | 0.041±0.068 | 0.007±0.015 | 0.3154 | 0.3649 |
| Terrisporobacter | 0.000±0.000 | 0.000±0.001 | 0.000±0.000 | 0.3893 | 0.3466 |
| Turicibacter | 0.043±0.033 | 0.068±0.018 | 1.369±1.017 | 0.2484 | 0.0337 |
| Unclassified | 67.492±4.060 | 12.510±9.044 | 29.447±8.249 | 0.0000 | 0.0244 |
| Veillonella | 0.003±0.005 | 2.932±1.387 | 2.920±5.775 | 0.0152 | 0.9969 |
| Vibrio | 0.000±0.001 | 0.000±0.000 | 0.000±0.001 | 0.2967 | 0.3466 |
| Victivallis | 0.000±0.000 | 0.001±0.001 | 0.000±0.001 | 0.3893 | 0.6801 |

Supplementary Table S4. Differences in microbial abundance at the class level in fecal samples.

|  | Water | DSS | Stigma+DSS | P-value  (Water versus DSS) | P-value  (DSS versus Stigma+DSS) |
| --- | --- | --- | --- | --- | --- |
| Actinobacteria | 0.389±0.462 | 0.138±0.118 | 0.062±0.029 | 0.2727 | 0.1990 |
| Alphaproteobacteria | 0.034±0.043 | 0.013±0.024 | 0.082±0.063 | 0.3663 | 0.0498 |
| Bacilli | 2.130±2.920 | 5.554±9.239 | 0.653±0.522 | 0.4522 | 0.2703 |
| Bacteroidia | 60.016±7.619 | 47.501±12.121 | 48.216±16.956 | 0.0864 | 0.9408 |
| Betaproteobacteria | 0.555±0.555 | 1.390±1.761 | 1.352±0.677 | 0.3416 | 0.9645 |
| Clostridia | 29.762±9.144 | 4.501±3.313 | 24.116±8.143 | 0.0004 | 0.0011 |
| Cyanobacteria | 0.000±0.000 | 0.000±0.000 | 0.000±0.001 | - | 0.3466 |
| Deferribacteres | 0.017±0.010 | 0.255±0.330 | 1.314±1.643 | 0.1449 | 0.1956 |
| Deltaproteobacteria | 1.070±0.434 | 0.885±0.492 | 4.216±2.704 | 0.5455 | 0.0266 |
| Epsilonproteobacteria | 0.485±0.272 | 2.229±1.437 | 13.238±9.754 | 0.0285 | 0.0371 |
| Erysipelotrichia | 2.146±1.128 | 2.551±1.626 | 2.821±1.921 | 0.6597 | 0.8162 |
| Flavobacteriia | 0.000±0.000 | 0.002±0.003 | 0.000±0.000 | 0.3466 | 0.3466 |
| Gammaproteobacteria | 0.017±0.019 | 31.901±18.713 | 0.361±0.290 | 0.0052 | 0.0055 |
| Lentisphaeria | 0.000±0.000 | 0.001±0.002 | 0.000±0.001 | 0.3466 | 0.6801 |
| Mollicutes | 0.241±0.427 | 0.002±0.003 | 0.012±0.016 | 0.2462 | 0.1957 |
| Negativicutes | 0.004±0.006 | 2.932±1.551 | 2.920±6.457 | 0.0029 | 0.9969 |
| Unclassified | 0.804±0.719 | 0.118±0.074 | 0.390±0.275 | 0.0668 | 0.0660 |
| Verrucomicrobiae | 2.331±1.831 | 0.028±0.021 | 0.247±0.459 | 0.0228 | 0.3175 |

Supplementary Table S5. Pearson correlation analysis between microbiota composition and concentrations of SCFAs (at the genus level, P＜0.05).

| Microbiota | SCFAs | Correlation Coefficient | P-value |
| --- | --- | --- | --- |
| Clostridium_III | butyrate | 0.7936 | 0.0004131 |
| Clostridium_IV | butyrate | 0.7863 | 0.0005085 |
| Saccharibacteria | butyrate | 0.7609 | 0.000987 |
| Clostridium_XlVa | butyrate | 0.7275 | 0.002115 |
| Parabacteroides | butyrate | -0.7002 | 0.003653 |
| Butyricimonas | butyrate | -0.7091 | 0.003073 |
| Bacteroides | butyrate | -0.8589 | 4.12E-05 |
| Bifidobacterium | isobutyrate | 0.8539 | 5.11E-05 |
| Dorea | isobutyrate | 0.8499 | 6.03E-05 |
| Alloprevotella | isobutyrate | 0.7853 | 0.0005223 |
| Megamonas | isobutyrate | 0.7835 | 0.0005484 |
| Butyrivibrio | isobutyrate | 0.7229 | 0.002326 |
| Rikenella | isovalerate | 0.8302 | 0.0001277 |
| Barnesiella | isovalerate | 0.7225 | 0.002346 |
| Clostridium_IV | isovalerate | 0.7138 | 0.002799 |
| Unclassified | isovalerate | 0.7102 | 0.003012 |
| Bacteroides | isovalerate | -0.7053 | 0.003315 |
| Bifidobacterium | propionate | 0.8300 | 0.0001287 |
| Alloprevotella | propionate | 0.7611 | 0.0009828 |
| Megamonas | propionate | 0.7484 | 0.001331 |
| Dorea | propionate | 0.7469 | 0.001376 |
| Butyrivibrio | propionate | 0.7237 | 0.00229 |
| Dorea | acetate | 0.7671 | 0.0008449 |
| Clostridium_IV | acetate | 0.7383 | 0.001673 |
| Bifidobacterium | acetate | 0.7292 | 0.002038 |
| Butyrivibrio | acetate | 0.7217 | 0.002388 |
| Pseudomonas | valerate | 0.7441 | 0.001468 |
| Clostridium_III | valerate | 0.7327 | 0.00189 |
